# CD33 Delineates Two Functionally Distinct NK Cell Populations Divergent in Cytokine Production and Antibody-Mediated Cellular Cytotoxicity

**DOI:** 10.3389/fimmu.2021.798087

**Published:** 2022-01-04

**Authors:** Maryam Hejazi, Congcong Zhang, Sabrina B. Bennstein, Vera Balz, Sarah B. Reusing, Melissa Quadflieg, Keven Hoerster, Stefan Heinrichs, Helmut Hanenberg, Sebastian Oberbeck, Marcus Nitsche, Sophie Cramer, Rita Pfeifer, Pranav Oberoi, Heiko Rühl, Johannes Oldenburg, Peter Brossart, Peter A. Horn, Florian Babor, Winfried S. Wels, Johannes C. Fischer, Nina Möker, Markus Uhrberg

**Affiliations:** ^1^ Institute for Transplantation Diagnostics and Cell Therapeutics, Heinrich-Heine University, Düsseldorf, Germany; ^2^ Miltenyi Biotec B.V. & Co. KG, Bergisch Gladbach, Germany; ^3^ Department of Pediatric Oncology, Hematology and Clinical Immunology, Center for Child and Adolescent Health, Medical Faculty, Heinrich-Heine University, Düsseldorf, Germany; ^4^ Institute for Transfusion Medicine, University Hospital Essen, University of Duisburg-Essen, Essen, Germany; ^5^ Department of Pediatrics III, University Children’s Hospital, University of Duisburg-Essen, Essen, Germany; ^6^ Department of Oncology, Hematology, Immuno-Oncology and Rheumatology, University Hospital of Bonn, Bonn, Germany; ^7^ Georg-Speyer-Haus, Institute for Tumor Biology and Experimental Therapy, Frankfurt am Main, Germany; ^8^ Institute of Experimental Hematology and Transfusion Medicine, University Hospital of Bonn, Bonn, Germany

**Keywords:** NK cell, CD33-CAR, cytokine production and cytotoxicity, RNAseq analysis, NK cell expansion

## Abstract

The generation and expansion of functionally competent NK cells *in vitro* is of great interest for their application in immunotherapy of cancer. Since CD33 constitutes a promising target for immunotherapy of myeloid malignancies, NK cells expressing a CD33-specific chimeric antigen receptor (CAR) were generated. Unexpectedly, we noted that CD33-CAR NK cells could not be efficiently expanded *in vitro* due to a fratricide-like process in which CD33-CAR NK cells killed other CD33-CAR NK cells that had upregulated CD33 in culture. This upregulation was dependent on the stimulation protocol and encompassed up to 50% of NK cells including CD56^dim^ NK cells that do generally not express CD33 *in vivo*. RNAseq analysis revealed that upregulation of CD33^+^ NK cells was accompanied by a unique transcriptional signature combining features of canonical CD56^bright^ (CD117^high^, CD16^low^) and CD56^dim^ NK cells (high expression of granzyme B and perforin). CD33^+^ NK cells exhibited significantly higher mobilization of cytotoxic granula and comparable levels of cytotoxicity against different leukemic target cells compared to the CD33^−^ subset. Moreover, CD33^+^ NK cells showed superior production of IFNγ and TNFα, whereas CD33^−^ NK cells exerted increased antibody-dependent cellular cytotoxicity (ADCC). In summary, the study delineates a novel functional divergence between NK cell subsets upon *in vitro* stimulation that is marked by CD33 expression. By choosing suitable stimulation protocols, it is possible to preferentially generate CD33^+^ NK cells combining efficient target cell killing and cytokine production, or alternatively CD33^−^ NK cells, which produce less cytokines but are more efficient in antibody-dependent applications.

## Introduction

Natural Killer (NK) cells are getting increasingly into the focus of allogeneic cell-based therapy due to their powerful effector mechanisms enabling eradication of tumor and virus-infected cells without eliciting graft-*versus*-host disease (1). Classically, circulating NK cells are divided into CD56^bright^ NK cells that are non-cytotoxic and primarily respond with IFNγ and TNFα production in response to exogenous cytokines and CD56^dim^ NK cells that are uniquely able to exert cytotoxic effector functions *via* CD16-mediated antibody-dependent cellular cytotoxicity (ADCC) as well as direct targeting of aberrant cells on the basis of missing self-recognition and expression of stress-induced ligands (2). Beyond these two major subsets, a multitude of less well-defined NK cell subpopulations with more subtle differences exist along a continuum of different functional states (3). Unfortunately, in NK cells stimulated and expanded *in vitro*, functional distinction of NK cell subsets on the basis of CD56 expression is not useful due to unspecific upregulation on virtually all NK cells. Similarly, other function-associated surface molecules are highly sensitive to shedding, such as CD16, or are quickly downregulated in culture, such as CD62L (4, 5). Thus, although it is apparent that the available protocols to expand NK cells do not lead to a homogenous pool of NK cells but rather result in a heterogeneous mixture of NK cell subsets with divergent functional capabilities, the tools to differentiate between functional subsets on the basis of surface molecules are scarce.

CD33 (siglec-3) is the smallest member of the sialic acid-binding immunoglobulin-like lectin (Siglec) family with only two Ig superfamily (IgSF)-like domains, a distal V domain mediating sialic acid binding and a membrane-proximal C2 domain (6). Besides the Siglec family-defining binding of CD33 to sialylated ligands, such as glycoproteins, glycolipids, and gangliosides, more specific protein ligands are currently unknown, with the exception of the recently defined complement component 1q (C1q) (7). The presence of an immunoreceptor tyrosine-based inhibitory motif (ITIM) and a second ITIM-like motif in the cytoplasmic domain suggests that CD33 is an inhibitory receptor, but the role of CD33 and its ITIM-mediated inhibition for regulation of the activation states of immune cells is elusive. Expression of CD33 is assumed to be largely restricted to the myeloid lineage, and with the exception of the CD56^bright^ NK cell subset, it seems to be absent from the lymphocytic lineages including mature T, B, and CD56^dim^ NK cells (8). CD33 is broadly expressed on various myeloid lineages and has gained therapeutic relevance as target on CD33-expressing myeloid and rare subsets of acute lymphoblastic leukemia (9).

Here, we studied the relevance of CD33 expression on *in vitro* stimulated NK cells, triggered by the initial observation of fratricide in cultures of CD33-CAR NK cells. We demonstrate that CD33 can be exploited to define two functionally distinct NK cell subsets representing CD33^+^ polyfunctional NK cells capable of strong cytokine production and target-based cytotoxicity and a CD33^−^ subset exhibiting efficient antibody-dependent cellular cytotoxicity (ADCC) due to strong expression of CD16. Notably, the CD33^+^ subset becomes highly abundant when using a commercially available medium-based protocol, whereas it remains only a minor subset when employing protocols using established stimulator cell lines. Thus, the frequency of the CD33 subset in the expanded NK cell product can be controlled *a priori* by choosing a suitable protocol for NK cell stimulation.

## Materials and Methods

### Human Samples

Buffy coats of healthy donors were kindly provided by the Blutspendezentrale at the University Hospital Düsseldorf. The protocol was accepted by the institutional review board at the University of Düsseldorf (study number 2019-383) and is in accordance with the Declaration of Helsinki. Peripheral blood mononuclear cells (PBMC) were isolated using density gradient centrifugation with Lymphocyte Separation Medium (PromoCell, Heidelberg, Germany).

### Flow Cytometry

The following fluorescence-labeled monoclonal antibodies (mAb) were used: CD3-FITC or PE/Cy5 (clone UCHT1), CD11c-FITC (3.9), CD16-APC/Cy7 (3G8), CD30-PE/Cy7 (BY88), CD33-PE, BV605 or PE/Cy5 (P67.6), CD56-PE/Dazzle™594 (N901), CD57-FITC (HCD57), CD62L-PE/Cy7 (DREG56), CD107-BV510 (H4A3), CD117-BV421 (104D2), KLRG1-APC/Fire 750 (SA231A2), PD1-APC/Cy7 (EH12-2H7), NKG2D-PE (1D11), NKp46-BV510 (9E2), NKp44-APC (P44-8), NKp30-BV785 (P30-15), Granzyme B-Pacific Blue (GB11), IFNγ PE/Cy7 (B27), Perforin-PE (dG9), TNFα PE/Dazzle™ 594 (all from Biolegend, CA, USA), CD3 PerCP-Vio700 (REA613), CD14-PerCP-Vio700 (REA599), CD33-APC, CD56 (REA196), anti-biotin-VioBright515 and 7-AAD (all Miltenyi Biotec), NKG2C-AF700 (134591) from R&D systems (MN, USA), and CD158b1,b2,j-PE/Cy5 (GL183), NKG2A-APC (Z199) purchased from Beckman Coulter (CA, USA). Flow cytometric analyses were performed on a CytoFLEX (Beckman Coulter) or MACSQuant 10 Analyzer (Miltenyi Biotec). Data analysis was performed on Kaluza 2.1.1 software.

### Cell Lines

The HLA class I-deficient target cell line K562 was grown in Dulbecco’s modified Eagle’s medium (DMEM) 4.5 g/L Glucose with L-Glutamine (Gibco, CA, USA) supplemented with 10% fetal bovine serum (FBS, FBS, Merck) and 1% Gentamycin. Human Burkitt lymphoma cell line Raji was cultivated in RPMI-1640 (Thermo Fisher Scientific, Waltham, MA, USA), 1% Penicillin/Streptomycin (P/S) (Gibco), and 10% FBS. K562-mb15-41BBL (10) (kindly provided by D. Campana, National University of Singapore) and K562-mb15-mb21-41BBL (11) were cultured in RPMI-1640, 1% Penicillin/Streptomycin, and 10% FBS. Human RS4;11 B cell precursor acute lymphoblastic leukemia (B-ALL) cells ectopically expressing human CD33 and GFP (RS4;11-CD33) or GFP only (RS4;11-GFP) were cultured in RPMI 1640 supplemented with 10% FBS and 2 mmol/L L-glutamine (Gibco). All cell lines used were free of mycoplasma.

### NK Cell Expansion

Isolation of pure NK cells was performed with MojoSort™ Human NK Cell Isolation Kit (Biolegend). PBMC or enriched NK cells were cultured in NK MACS medium [1% NK MACS supplement, 5% human AB serum (Sigma Aldrich), 500 U/ml IL-2 (Proleukin), and 25ng/ml IL-15 (Miltenyi Biotec)]. NK cell expansion with stimulator cells was performed as previously described (10). Briefly, PBMCs (1.5×10^6^) were incubated in 24-well plates with 1×10^6^ irradiated (40 Gy) K562-mb15-41BBL cells or K562-mb15-mb21-41BBL in SCGM CellGro Medium (CellGenix, Freiburg, Germany) supplemented with 10% FBS and 1% P/S and 40 U/ml human IL-2 (Proleukin).

### Chimeric Antigen Receptor NK Cells

CAR constructs were designed *in silico* and consist of a human GM-CSFRα signal peptide, an antigen-specific scFv derived from Gemtuzumab (CD33-CAR) or FMC63 (CD19-CAR), a CD8α hinge and transmembrane (TM) domain, followed by 4-1BB and CD3ζ intracellular (IC) domains. Codon-optimized CAR constructs were inserted into a third-generation lentiviral plasmid backbone (Lentigen Technology Inc., Gaithersburg, MD, USA) under control of a human EF-1α promoter. Baboon envelope (BaEV) pseudotyped lentiviral vectors (LV) containing supernatants were generated by transient transfection of HEK 293T cells, as previously described (12). NK cell enrichment was performed with NK cell isolation kit for human cells (Miltenyi Biotec). NK cells stimulated with NK MACS medium and 80 ng/ml of IL-1ß (day 2) were transduced with BaEV-LV encoding CD33-CAR or CD19-CAR constructs in the presence of 10 μg/ml Vectofusin-1 for 24 h, after 2 h spinoculation at 400 × g, 32°C (13). CAR expression was flow cytometrically evaluated using biotinylated human recombinant CD33-Fc protein (R&D Systems) or CD19 detection reagent (Miltenyi Biotec), respectively, followed by VioBright515-conjugated antibiotin antibody (Miltenyi Biotec).

### RNA Sequencing

CD33^+^ and CD33^−^ NK cells were flow cytometrically sorted (MoFlo XDP, Beckman Coulter) and stored in TRIzol Reagent (Invitrogen, Carlsbad, CA, USA) for extraction of total RNA. Reverse transcription and library production were performed in the NGS Integrative Genomics (NIG) facility in Göttingen, Germany, with an Illumina Truseq RNA preparation kit. Sequencing of the libraries was performed on an Illumina HiSeq4000 (single-read 1 × 50 bp). Sequence reads were mapped to the human genome (hg38) and analyzed using DESeq2 software (v1.26.0) as described previously (14, 15). Heatmaps and volcano plots were performed with R packages pretty heatmap (pheatmap) (v1.0.12) (16) and EnhancedVulcano (1.4.0) (17), respectively.

### NK Cell Function

For analysis of degranulation and cytokine production, NK cells and K562 target cells were mixed at an effector/target (E/T) ratio of 1:1 in a volume of 200 µl in a 96-well plate (round bottom). CD107a mAb was added prior to incubation. To determine spontaneous degranulation or cytokine production, control samples without target cells were included. After 1 h incubation time, 2 µl of 2 mM Monensin and 2 µl Brefeldin (1000×, Biolegend) were added and samples incubated for further 4 h. Subsequently, cells were stained for selected surface markers. Interferon-γ and TNF-α were intracellularly stained following treatment with fixation and permeabilization buffer (Biolegend). For cytotoxicity analysis, K562 target cells were stained with 5 mM CFDA-SE (Invitrogen) and mixed with NK cells at various E/T ratios. After 5 h incubation, propidium iodide (PI, Biolegend) was added shortly before flow cytometric analysis to quantify the viable target cells as described previously (18). In some experiments, cytotoxicity of CAR NK cells was assessed against GFP-expressing tumor cells. In order to determine CD33-CAR-induced NK cell fratricide, *in vitro* expanded NK cells were labeled with 1 μM CellTrace Violet (CV, Thermo Fisher Scientific, Waltham, MA, USA) and cocultured with unlabeled CD33-CAR NK cells at various E/T ratios for 24 h. For ADCC analysis, 1 µg/ml of rituximab (Truxima^®^, Celltrion Healthcare, South Korea) was added directly to the co-culture of NK cells and Raji target cells (ratio 1:1). NK cells without Raji and NK cells with Raji but without Rituximab served as controls.

### Statistical Analyses

Normal distribution of the data was calculated with Shapiro-Wilk normality test. Depending on normality, paired/unpaired t-test or Mann-Whitney test was performed. The difference between more than two groups was analyzed with 1-Way ANOVA. All analyses were done using GraphPad Prism 8.0.0 (GraphPad Software, CA, USA).

## Results

### CD33^+^ NK Cells Are Recognized and Eliminated by CD33-CAR NK Cells

**Figure 1:**
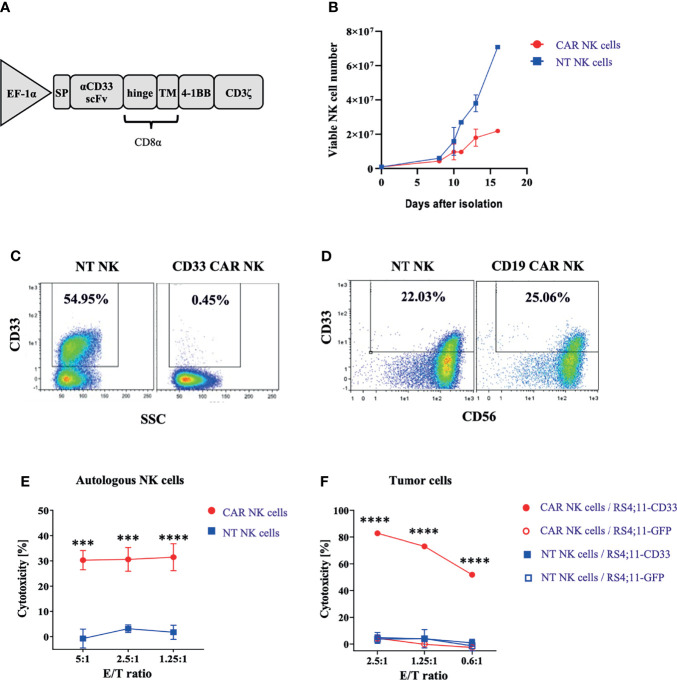
CD33-CAR-mediated NK cell fratricide. **(A)** CD33-CAR construct design. **(B)** *In vitro* proliferation of non-transduced (NT) NK cells and CD33-CAR NK cells after isolation. CD33 expression on **(C)** CD33-CAR NK cells and **(D)** CD19-CAR NK cells was determined by flow cytometry (day 14) compared with NT NK cells **(E)** CD33-CAR-induced NK cell fratricide with *ex vivo* expanded autologous NK cells as targets. NT NK cells were included as control. **(F)** Cytotoxicity of CD33-CAR NK cells against CD33-expressing RS4;11 tumor cells. NT NK cells and CD33-negative RS4;11-GFP cells were included as controls. Data were analyzed by two-tailed unpaired t-test ***p < 0.001; ****p < 0.0001.

Our interest in CD33^+^ NK cells was fueled by an observation made during the generation and expansion of CD33-specific CAR NK cells for therapy of myeloid leukemia. To this end, PBMC-derived and magnetically enriched NK cells were transduced with a Baboon envelope (BaEV)-pseudotyped lentiviral vector (LV) carrying a CD33-specific CAR. The construct consisted of an antigen-specific scFv antibody domain derived from the CD33-specific mAb Gemtuzumab, CD8α hinge and transmembrane (TM) domains, and 4-1BB and CD3ζ intracellular (IC) domains (**Figure 1A**). Following lentiviral transduction, CD33-CAR NK cells were stimulated using NK MACS medium, supplemented with IL-2 and IL-15, for 2 weeks. As shown in **Figure 1B**, NK cells expanded much less in the transduced setting compared to non-transduced NK cells. Flow cytometric analyses revealed that this was due to selective depletion of CD33^+^ NK cells, whereas CD33^−^ NK cells remained unaffected (**Figure 1C**). Depletion of CD33^+^ NK cells was dependent on the presence of CD33-CAR NK cells since in non-transduced controls, CD33^+^ NK cells were present at high frequencies (>50%) (**Figure S1**). Furthermore, when employing control CAR constructs with specificity for CD19 instead of CD33 (but otherwise identical features), no such effects on the frequency of CD33^+^ NK cells were noted, and no overt inhibition of NK cell expansion was observed for CD19-CAR-transduced compared to non-transduced NK cells (**Figure 1D**). The data suggested that CD33^+^ NK cells arising in the cultures were targets for CD33-CAR NK cells. To more closely look into this possibility, we analyzed the cytotoxic activity of CD33-CAR NK cells against autologous NK cells, labeled with a cell tracing fluorescent reagent. Indeed, CD33-CAR NK cells exhibited substantial cytotoxicity against autologous NK cells that were stimulated for 2 weeks in order to induce CD33 expression, whereas non-transduced NK cells did not show cytotoxicity against the same autologous NK cell targets (**Figure 1E**). Finally, the specificity of CD33-CAR NK cells was tested against CD33^+^ tumor cells: the RS4;11 tumor cell line was killed by CD33-CAR NK cells with high efficiency when expressing CD33, whereas a variant that did not express CD33 (GFP^+^RS4;11) was not recognized (**Figure 1F**). Similarly, non-transduced NK cells did not kill the tumor cells regardless of CD33 expression due to natural resistance of RS4;11 cells to NK cell-mediated killing. Thus, the observed killing of NK cells by other autologous NK cells, referred to as fratricide, was due to recognition of CD33 by the respective CD33-CAR NK cells.

### CD33 Expression in Culture Is Primarily Due to Upregulation on CD56^dim^ NK cells and Depends on the Stimulation Protocol

**Figure 2:**
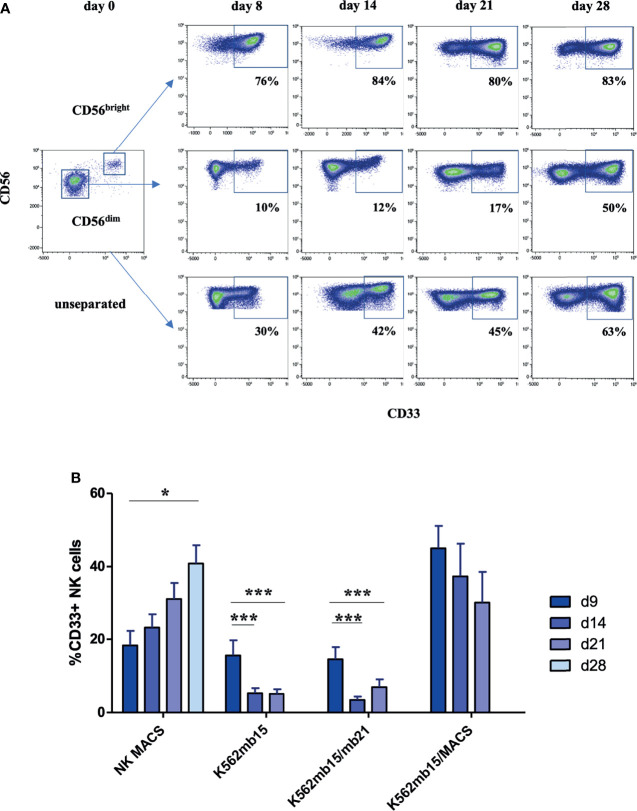
Upregulation of CD33 on CD56^dim^ NK cells depends on stimulation protocol. **(A)** CD56^dim^ and CD56^bright^ NK cells were enriched by flow cytometric cell sorting and subsequently cultured in NK MACS medium for 28 days. Unsorted NK cells served as control. Flow cytometric dot plots of one representative donor of three show CD33 expression on CD56^dim^, CD56^bright^, and unseparated NK cells cultured in NK MACS medium on days 0, 8, 14, 21, and 28. **(B)** CD33 expression on NK cells stimulated with different stimulatory protocols using NK MACS medium (n=10), K562-mb15-41BBL (n=10), or K562-mb15-mb21-41BBL (n=8) cells and combination of K562-mb15-41BBL and NK MACS medium (n=3) on days 0, 8, 14, 21, and 28. Data were analyzed by 1-way ANOVA, *p < 0.05; ***p < 0.001.

CD33 is expressed *in vivo* on CD56^bright^ but not CD56^dim^ NK cells (8). We were thus wondering if the CD33^+^ NK cells developing in culture were also primarily due to expansion of CD56^bright^ NK cells. We thus flow cytometrically sorted the two respective CD56 subsets to high purity from peripheral blood and cultured them separately for 4 weeks in NK MACS medium. As shown in **Figure 2A**, both subsets contributed to the expansion of the CD33^+^ subset: whereas in CD56^bright^ NK cells the large majority of cells maintained CD33 expression, in CD56^dim^ NK cells the frequency of CD33 increased over time from basically no expression at the beginning of culture to around 50% at day 28 as shown in the exemplary experiment in **Figure 2A**. The CD56^dim^ data largely resembled the kinetics of CD33 expression in the control setup using unseparated NK cells, which was expected since CD56^dim^ NK cells typically constitute more than 90% of all NK cells and thus are the dominant subset contributing to the increase in CD33^+^ cells observed in the cultures (**Figure 2A**).

Of note, in about 10% of samples, CD33 expression remained low during stimulation, and this was accompanied by a lack of CD33 expression on CD56^bright^ NK cells *in vivo* (**Figure S2A**). Subsequent search for a putative underlying genetic polymorphism by targeted sequencing of CD33 (**Figures S2B, C**) revealed that all samples, which did not express CD33 on the CD56^bright^ subset and failed to upregulate CD33 during culture, carried a previously described single nucleotide polymorphism (SNP, rs12459419 C>T) associated with skipping of exon 2 encoding the IgV domain of CD33 due to alternative splicing (19). The SNP occurred either in homozygous configuration or in one case in combination with a known, more rarely occurring null allele (**Figure S2C**). Since the IgV domain encodes the epitope recognized by the CD33-specific antibody used in the CAR (and other commercially available mAbs), failure to detect CD33 expression in culture seems to be largely due to this splicing polymorphism (20). This notion is also compatible with the allelic frequency of the SNP (rs12459419), which is found in homozygous configuration in approximately 10% of the Caucasoid population (https://gnomad.broadinstitute.org/variant/19-51728477-C-T?dataset=gnomad_r2_1).

Besides NK MACS medium, another established method for expansion of primary NK cells utilizes stimulator cells expressing the ligand for 4.1BB (CD137) together with a membrane-bound version of IL-15, IL-21, or both (10, 11). In order to understand how far the particular stimulatory protocol influences the upregulation of CD33, we comparatively analyzed the different NK cell stimulation protocols side by side. As shown in **Figure 2B**, upregulation of CD33 was significantly stronger when using NK MACS medium compared to protocols using K562 stimulator cells expressing 4.1BB and membrane-bound IL-15 (K562-mb15-41BBL) or additionally membrane-bound IL-21 (K562-mb15-mb21-41BBL). Analysis of the kinetics revealed that whereas NK MACS medium led to a continuous increase in CD33 expression over time as already outlined above, the K562-based protocols exhibited an initial moderate increase to 10–20% CD33^+^ cells before significantly decreasing to <5% at day 14 and maintaining low levels until day 21 (the timepoint when NK cells are harvested according to the protocol). Mechanistically, upregulation of CD33 expression could be either a default process during *in vitro* stimulation of NK cells that is eventually inhibited by the stimulator cell lines or the NK MACS medium could provide a soluble stimulus for CD33 expression. We thus combined both protocols by using K562-mb15-41BBL cells together with NK MACS medium and found that CD33 expression is rescued by addition of the NK MACS medium (**Figure 2B**), suggesting that a soluble medium-derived factor is involved in induction of CD33 expression on NK cells.

### CD33 Delineates Two NK Cell Subsets With Unique Transcriptional Signatures

**Figure 3:**
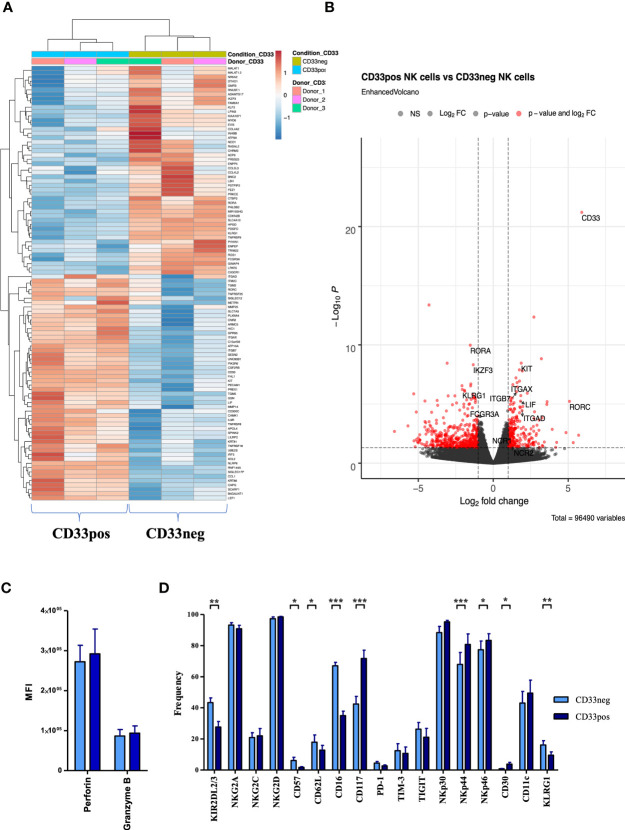
Different transcriptional and phenotypical characteristics of CD33^−^ and CD33^+^ NK cells. **(A)** CD33^−^ and CD33^+^ NK cells were sorted on day 21 following culture with NK MACS medium (n=3). RNA sequencing was performed on the Illumina platform. The heatmap illustrates the top 100 differentially expressed genes between CD33^−^ and CD33^+^ NK cells. **(B)** The volcano plot indicates the RNA sequencing data based on 55,394 genes by plotting the logarithm of the fold change between CD33^−^ and CD33^+^ NK cells on the x axis and the negative logarithm of the p value on the y axis. The dashed lines indicate p values equaling 0.05. Red points represent the genes with highest statistical significance of fold change. Top genes of interest are labeled. Stimulated NK cells with NK MACS medium (days 14–21) were analyzed for **(C)** intracellular perforin and granzyme B (n=5) or **(D)** for the cell-surface expression of the indicated molecules (n=17) by flow cytometry. Expression was compared between CD33^-^ and CD33^+^ NK cells. Statistical significance was determined by paired t-test, *p < 0.05; **p < 0.01; ***p < 0.001.

In order to gain more insights into the phenotypic and transcriptional characteristics of CD33^+^ and CD33^−^ NK cell subsets, PBMC-derived NK cells were cultured for 2 weeks in NK MACS medium and subsequently sorted by flow cytometry into the two CD33 subsets for further analysis by RNAseq. Heatmap analyses demonstrated that the two CD33 subsets have divergent transcriptional signatures leading to separate clustering of CD33^+^ and CD33^−^ subsets (**Figure 3A**). More than 200 genes (adjusted p < 0.05) were found to be differentially expressed between the two subsets (**Figure S3**). Among the most significant differences were the transcription factors RORA, encoding the RAR-related orphan receptor α (RORα), which was upregulated in CD33^−^ NK cells, and RORC, encoding RORγT, which was more abundant in CD33^+^ NK cells (**Figure 3B**). RORα and RORγt are well-known for their involvement in regulation of innate lymphoid cell (ILC) 2 and ILC3 development, respectively, but their role in NK cell development is currently unknown (21). The third highly significant difference in TF expression was represented by the Ikaros family member Aiolos (IKZF3), which was downregulated in CD33^+^ NK cells. Other significantly overexpressed genes in CD33^+^ NK cells are receptors involved in cell-cell interaction such as the integrins ITGAX (CD11c), ITGAD (CD11d), and ITGB7. This is compatible with analysis of the underlying biological pathways showing highest significance for differences in the adhesion pathway (**Figure S4**).

The expression differences between CD33^+^ and CD33^−^ NK cells for several genes encoding cell surface receptors such as abundant transcripts for CD117 (c-kit) and low transcripts of KLRG1 and CD16 (FCGR3A) (**Figures 3A, B**) were reminiscent of the differences between CD56^bright^ and CD56^dim^ NK cells (22), suggesting that CD33^+^ NK cells might be more similar to CD56^bright^ NK cells. However, within principal component analysis (PCA), CD33^+^ NK cells were not closer related to CD56^bright^ NK cells than CD33^−^ NK cells (data not shown). Moreover, this similarity did not extend to key cytotoxic molecules such as perforin and granzyme B, which were strongly expressed in both subsets on the mRNA (data not shown) and protein level (**Figure 3C**). Next, we analyzed surface expression of a panel of typical NK cell-related molecules: besides verifying the transcriptional differences in CD16 and CD117 expression, we noted a higher expression of KIR receptors and a small subset of CD33^−^ cells that expressed CD57 and KLRG1, two markers of terminally differentiated NK cells (23). The expression of natural cytotoxicity receptors NKp44 and NKp46 was significantly higher in CD33^+^ NK cells, whereas no significant differences were found for NKp30, the lectin-like family of NK cell receptors NKG2A, C, and D, or the checkpoint inhibitors PD-1, TIM3, and TIGIT (**Figure 3D**). Finally, we compared the data generated in small-scale tissue culture (24-well plate) to a single large-scale experiment using the CliniMACS Prodigy system. The phenotypes of CD33^+^ and CD33^−^ NK cell subsets generated with the Prodigy platform were highly similar to those seen in 24-well plate cultures, demonstrating that similar kinds of CD33^+^ and CD33^−^ NK cell subsets are generated when transferring the small-scale conditions to a large-scale GMP-compatible setup (**Figure S5A**). Only CD62L, a marker of naïve NK cells, appeared to be somewhat more frequent in the CD33^+^ subset in the large-scale experiment.

### CD33^+^ NK Cells Are Polyfunctional, Combining Strong Cytokine Production and Cytotoxicity

**Figure 4:**
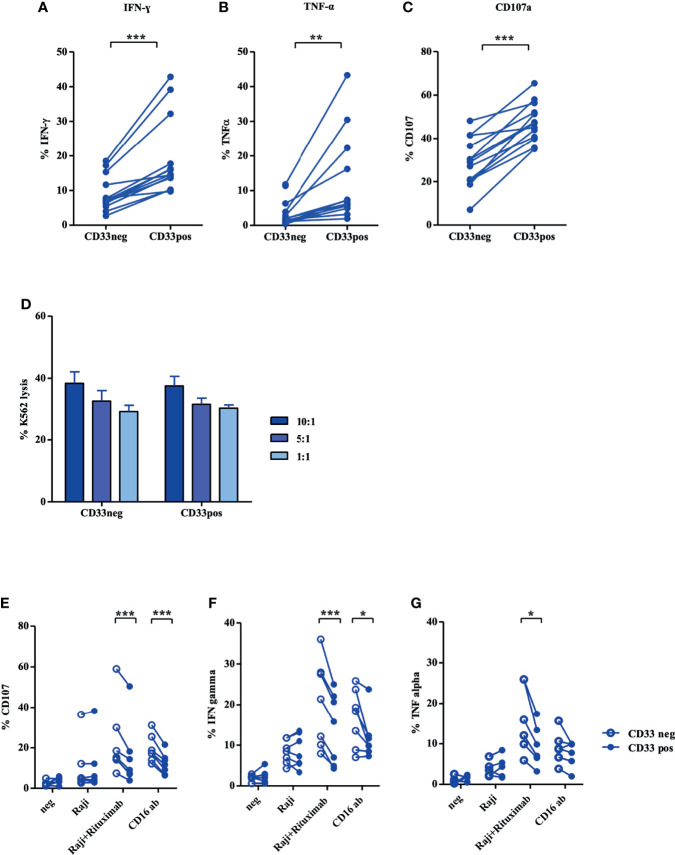
CD33^+^ NK cells display strong functionality in both cytokine production and cytotoxicity. Stimulated NK cells with NK MACS medium (day 14) were incubated with K562 cells at an effector/target ratio of 1:1. Intracellular **(A)** IFN-γ and **(B)** TNF-α production and **(C)** CD107a mobilization were evaluated by flow cytometry (n=13). **(D)** CFDA-SE stained K562 cells were incubated with CD33^+^ and CD33^−^ NK cells in effector/target ratios of 10:1, 5:1, and 1:1 (n=10). For ADCC, NK cells stimulated in NK MACS medium (day 14) were incubated with/without Raji and Rituximab, and **(E)** CD107a expression and intracellular **(F)** IFN-γ and **(G)** TNF-α production were measured by flow cytometry (n=8). Statistical significance was determined by paired t-test, *p < 0.05; **p < 0.01; ***p < 0.001.

Next, we assessed how far the distinct transcriptional and phenotypic properties translate into functional differences between the two NK cell subsets. As shown in **Figure 4**, a significantly higher frequency of CD33^+^ cells produced IFNγ and TNFα in response to K562 target cells compared to the CD33^−^ subset (**Figures 4A, B**). Notably, CD33^+^ NK cells also exhibited a higher frequency of CD107^+^ cells, reflecting more effective mobilization of cytotoxic granules to the cell surface (**Figure 4C**). However, when measuring direct cytotoxicity against K562 cells, both subsets showed comparable lysis over a range of effector/target ratios (**Figure 4D**). Similar results were obtained when NK cells were expanded with the CliniMACS Prodigy system (**Figure S5B**). Finally, we assessed antibody-dependent cellular cytotoxicity (ADCC) employing the NK cell-resistant CD20^+^ target cell line Raji in combination with the therapeutic anti-CD20 reagent Rituximab, which is a human IgG1 mAb that binds with its Fc part to the CD16 receptor on NK cells. In accordance with the significantly higher expression of CD16, CD33^−^ NK cells exhibited stronger mobilization of cytotoxic granules and higher cytokine responses to Raji compared to CD33^+^ NK cells, with IFNγ showing more significant differences than TNFα (**Figures 4E–G**).

## Discussion

The expression of CD33 is largely restricted to the myeloid lineage and recently gained much interest as a target for immunotherapy of AML and other CD33-expressing malignancies, initially with the antibody-drug conjugate Gemtuzumab Ozagamicin, followed by bispecific antibody conjugates, antibody-cytokine conjugates, and lately CD33-CAR T cells (9, 24–26). Unexpectedly, we found that CD33 is upregulated *in vitro* on a significant subset of NK cells. Although *in vivo* CD33 expression is restricted to CD56^bright^ NK cells, which are a subset of non-cytotoxic, rather immature NK cells, this was not the case *in vitro*: CD33 could be efficiently induced on purified CD56^dim^ NK cells leading to frequencies of CD33^+^ NK cells up and above 50% with NK MACS medium. The present work suggests that CD33 constitutes a marker to distinguish between functionally divergent NK cell subsets *in vitro*. Expression of CD33 defines a polyfunctional NK cell subset combining cytotoxic and cytokine effector functions. On the other hand, due to lower expression of CD16, CD33^+^ NK cells are less efficient in ADCC compared to CD33^−^ NK cells, which in turn produce significantly less cytokines. Interestingly, CD33 seems to demarcate two separate cell states, which are either positive or negative with very few NK cells expressing intermediate levels of CD33 (see also **Figures 2**, **3**). Comparative transcriptional analysis by RNAseq revealed that the transcriptional programs of the two subsets are not simply mirroring CD56^bright^ and CD56^dim^ subsets but constitute quite independent cellular entities compatible with their unique functional properties, making both subsets potentially interesting tools for cancer therapy. It remains to be determined which of the differentially expressed TFs are involved in orchestrating these transcriptional changes, e.g., RORα and RORγT, which are so far described as master regulators of ILC2 and ILC3 development, respectively (21). Furthermore, downregulation of the Ikaros family member Aiolos in CD33^+^ NK cells is interesting in this context since it was previously reported to be involved in shaping of the final NK cell maturation program in an Aiolos-deficient mouse model (27).

The upregulation of CD33 on NK cells *in vitro* is significant and strong enough to mediate recognition and subsequent fratricide by CD33-CAR NK cells. Whereas this principally hampers their expansion for cell therapeutic purposes, our study suggests that the problem can be circumvented in several ways: firstly, about 10% of the Caucasoid population are homozygous for an SNP (rs12459419) that leads to efficient alternative splicing and skipping of the IgV domain of CD33, which is the binding site for the CAR used in this study (19, 20). Due to the lack of the CD33 target site, those donors could be selected for expansion of CD33-CAR NK cells without inhibition by fratricide and stored for further clinical use such as allogeneic therapy of AML. Of note, due to the lack of GvH disease, application of CAR NK cells in the allogeneic setting constitutes a major advantage compared to CAR T cell therapies, which are presently only applicable in the autologous setting (28). Moreover, upregulation of CD33 was largely restricted to NK MACS medium, whereas an alternative protocol for expansion of NK cells based on K562 stimulator cells, which is already part of NK cell-based clinical protocols (29), did only lead to a transient wave of CD33 expression. Whether fading of the CD33 subset with stimulator cell-based protocols is due to downregulation of CD33 or possibly outperformance by CD33^−^ NK cells is currently unknown. In any case, by adding NK MACS medium to stimulator cell cultures, expression of CD33 is again induced, suggesting a yet undefined component in the medium that supports expansion of the CD33^+^ subset.

The biological role of CD33 expression on CD56^bright^ NK cells is currently unclear. Generally, as a member of the Siglec family, CD33 is able to recognize sialylated ligands, but so far, no specific cell-bound glycoproteins or other glycosylated ligands were identified that are preferentially recognized by NK cells. In functional terms, the CD33 receptor contains intracytoplasmic ITIM motifs, and it was previously shown that crosslinking of CD33 leads to inhibition of effector functions (30). Notably, Siglecs were shown to bind not only in *trans* but also in *cis* to cell-bound ligands, which might lead to a constitutive inhibitory state (30). However, our study clearly shows that CD33^+^ NK cells have polyfunctional characteristics marked by strong cytokine production and efficient killing. The association of ITIM-containing inhibitory receptors with gain of function is not without precedent in NK cells, since expression of ITIM-containing inhibitory KIR receptors mediate licensing of NK cells, an educational process associated with a strong gain of function (31). Similarly to CD33, inhibitory KIRs are providing a constitutive inhibitory state since they bind to ubiquitous HLA class I molecules present on all healthy nucleated cells. Moreover, both KIR and the Siglec family are not only structurally related but are also genetically linked in the extended leukocyte receptor complex (LRC) on chromosome 19q13.4 (32). At this time, it can only be speculated whether these similarities including the common signal transduction *via* SHP1 and SHP2 could be an indicator for a similar licensing-like function of CD33.

Altogether, expression of CD33 delineates a novel transcriptional and functional dichotomy that arises during *in vitro* expansion of NK cells. In terms of clinical translation, analysis of CD33 expression might give simple guidance on the composition of NK cell products during the *in vitro* expansion process for clinical use, with increased cytokine production and mobilization of cytotoxic granula on CD33^+^ cells on the one hand and superior CD16-mediated functions on CD33^−^ NK cells on the other hand.

## Data Availability Statement

The data presented in the study are deposited in NCBI Project ID: PRJNA777044 (http://www.ncbi.nlm.nih.gov/bioproject/777044).

## Ethics Statement

The studies involving human participants were reviewed and approved by Ethikkommission Düsseldorf, Medical Faculty (study number 2019-383). The patients/participants provided their written informed consent to participate in this study.

## Author Contributions

MH, CZ, MU, and NM conceived and planned the experiments. MH, CZ, KH, SO, and VB carried out the experiments. MH and SBB performed calculations. MH, CZ, SB, and VB conducted data analysis and interpretation. MU supervised the project. MH and MU wrote the manuscript. MU, SB, WW, and PH edited the manuscript. MN, SC, and RP provided data. SR, MQ, SH, HH, PO, HR, JO, PB, PH, FB, WW, and JF provided critical feedback and commented on the manuscript. All authors contributed to the article and approved the submitted version.

## Funding

This work was conducted in the framework of the iCAN33 project, funded by the European Regional Development Fund NRW (ERDF, German EFRE) 2014-2020.

## Conflict of Interest

CZ, MQ, MN, SC, RP, and NM are employees of Miltenyi Biotec.

The remaining authors declare that the research was conducted in the absence of any commercial or financial relationships that could be construed as a potential conflict of interest.

## Publisher’s Note

All claims expressed in this article are solely those of the authors and do not necessarily represent those of their affiliated organizations, or those of the publisher, the editors and the reviewers. Any product that may be evaluated in this article, or claim that may be made by its manufacturer, is not guaranteed or endorsed by the publisher.

## References

[1] Miller JS Lanier LL. Natural Killer Cells in Cancer Immunotherapy. Annu Rev Cancer Biol (2019) 3:77–103. doi: 10.1146/annurev-cancerbio-030518-055653

[2] Caligiuri MA. Human Natural Killer Cells. Blood (2008) 112(3):461–9. doi: 10.1182/blood-2007-09-077438 PMC2481557 18650461

[3] Freud AG Mundy-Bosse BL Yu J Caligiuri MA. The Broad Spectrum of Human Natural Killer Cell Diversity. Immunity (2017) 47(5):820–33. doi: 10.1016/j.immuni.2017.10.008 PMC5728700 29166586

[4] Romee R Foley B Lenvik T Wang Y Zhang B Ankarlo D. NK Cell CD16 Surface Expression and Function is Regulated by a Disintegrin and Metalloprotease-17 (ADAM17). Blood (2013) 121(18):3599–608. doi: 10.1182/blood-2012-04-425397 PMC3643761 23487023

[5] Moustaki A Argyropoulos KV Baxevanis CN Papamichail M Perez SA. Effect of the Simultaneous Administration of Glucocorticoids and IL-15 on Human NK Cell Phenotype, Proliferation and Function. Cancer Immunol Immunother (2011) 60(12):1683–95. doi: 10.1007/s00262-011-1067-6 PMC11029608 21706285

[6] Crocker PR Paulson JC Varki A. Siglecs and Their Roles in the Immune System. Nat Rev Immunol (2007) 7(4):255–66. doi: 10.1038/nri2056 17380156

[7] Son M Diamond B Volpe BT Aranow CB Mackay MC Santiago-Schwarz F. Evidence for C1q-Mediated Crosslinking of CD33/LAIR-1 Inhibitory Immunoreceptors and Biological Control of CD33/LAIR-1 Expression. Sci Rep (2017) 7(1):270. doi: 10.1038/s41598-017-00290-w 28325905 PMC5412647

[8] Handgretinger R Schafer HJ Baur F Frank D Ottenlinger C Buhring HJ. Expression of an Early Myelopoietic Antigen (CD33) on a Subset of Human Umbilical Cord Blood-Derived Natural Killer Cells. Immunol Lett (1993) 37(2-3):223–8. doi: 10.1016/0165-2478(93)90034-Y 7505004

[9] Lichtenegger FS Krupka C Haubner S Kohnke T Subklewe M. Recent Developments in Immunotherapy of Acute Myeloid Leukemia. J Hematol Oncol (2017) 10(1):142. doi: 10.1186/s13045-017-0505-0 28743264 PMC5526264

[10] Fujisaki H Kakuda H Shimasaki N Imai C Ma J Lockey T. Expansion of Highly Cytotoxic Human Natural Killer Cells for Cancer Cell Therapy. Cancer Res (2009) 69(9):4010–7. doi: 10.1158/0008-5472.CAN-08-3712 PMC2716664 19383914

[11] Oberoi P Kamenjarin K Ossa JFV Uherek B Bonig H Wels WS. Directed Differentiation of Mobilized Hematopoietic Stem and Progenitor Cells Into Functional NK Cells With Enhanced Antitumor Activity. Cells (2020) 9(4):811. doi: 10.3390/cells9040811 PMC7226771 32230942

[12] Girard-Gagnepain A Amirache F Costa C Levy C Frecha C Fusil F. Baboon Envelope Pseudotyped LVs Outperform VSV-G-LVs for Gene Transfer Into Early-Cytokine-Stimulated and Resting HSCs. Blood (2014) 124(8):1221–31. doi: 10.1182/blood-2014-02-558163 24951430

[13] Bari R Granzin M Tsang KS Roy A Krueger W Orentas R. A Distinct Subset of Highly Proliferative and Lentiviral Vector (LV)-Transducible NK Cells Define a Readily Engineered Subset for Adoptive Cellular Therapy. Front Immunol (2019) 10:2001. doi: 10.3389/fimmu.2019.02001 31507603 PMC6713925

[14] Bennstein SB Weinhold S Manser AR Scherenschlich N Noll A Raba K. Umbilical Cord Blood-Derived ILC1-Like Cells Constitute a Novel Precursor for Mature KIR(+)NKG2A(-) NK Cells. Elife (2020) 9:e55232. doi: 10.7554/eLife.55232 32657756 PMC7358013

[15] Bennstein SB Scherenschlich N Weinhold S Manser AR Noll A Raba K. Transcriptional and Functional Characterization of Neonatal Circulating ILCs. Stem Cells Transl Med (2021) 10:867–82. doi: 10.1002/sctm.20-0300 PMC8133339 33475258

[16] Kolde R. Pretty Heatmaps (2015). Available at: https://CRANR-projectorg/package=pheatmap.

[17] Glighe K Rana S Lewis M. EnhancedVolcano: Publication-Ready Volcano Plots With Enhanced Colouring and Labeling (2019). Available at: https://githubcom/kevinblighe/EnhancedVolcano.

[18] Hejazi M Manser AR Frobel J Kundgen A Zhao X Schonberg K. Impaired Cytotoxicity Associated With Defective Natural Killer Cell Differentiation in Myelodysplastic Syndromes. Haematologica (2015) 100(5):643–52. doi: 10.3324/haematol.2014.118679 PMC4420213 25682594

[19] Lamba JK Chauhan L Shin M Loken MR Pollard JA Wang YC. CD33 Splicing Polymorphism Determines Gemtuzumab Ozogamicin Response in *De Novo* Acute Myeloid Leukemia: Report From Randomized Phase III Children's Oncology Group Trial Aaml0531. J Clin Oncol (2017) 35(23):2674–82. doi: 10.1200/JCO.2016.71.2513 PMC5549451 28644774

[20] Gbadamosi MO Shastri VM Hylkema T Papageorgiou I Pardo L Cogle CR. Novel CD33 Antibodies Unravel Localization, Biology and Therapeutic Implications of CD33 Isoforms. Future Oncol (2021) 17(3):263–77. doi: 10.2217/fon-2020-0746 PMC10621775 33356566

[21] Vivier E Artis D Colonna M Diefenbach A Di Santo JP Eberl G. Innate Lymphoid Cells: 10 Years on. Cell (2018) 174(5):1054–66. doi: 10.1016/j.cell.2018.07.017 30142344

[22] Michel T Poli A Cuapio A Briquemont B Iserentant G Ollert M. Human CD56bright NK Cells: An Update. J Immunol (2016) 196(7):2923–31. doi: 10.4049/jimmunol.1502570 26994304

[23] Bjorkstrom NK Riese P Heuts F Andersson S Fauriat C Ivarsson MA. Expression Patterns of NKG2A, KIR, and CD57 Define a Process of CD56dim NK-Cell Differentiation Uncoupled From NK-Cell Education. Blood (2010) 116(19):3853–64. doi: 10.1182/blood-2010-04-281675 20696944

[24] Sarhan D Brandt L Felices M Guldevall K Lenvik T Hinderlie P. 161533 TriKE Stimulates NK-Cell Function to Overcome Myeloid-Derived Suppressor Cells in MDS. Blood Adv (2018) 2(12):1459–69. doi: 10.1182/bloodadvances.2017012369 PMC6020813 29941459

[25] Kenderian SS Ruella M Shestova O Klichinsky M Aikawa V Morrissette JJ. CD33-Specific Chimeric Antigen Receptor T Cells Exhibit Potent Preclinical Activity Against Human Acute Myeloid Leukemia. Leukemia (2015) 29(8):1637–47. doi: 10.1038/leu.2015.52 PMC4644600 25721896

[26] Reusing SB Vallera DA Manser AR Vatrin T Bhatia S Felices M. CD16xCD33 Bispecific Killer Cell Engager (BiKE) as Potential Immunotherapeutic in Pediatric Patients With AML and Biphenotypic ALL. Cancer Immunol Immunother (2021) 70:3701–8. doi: 10.1007/s00262-021-03008-0 PMC8571204 34398302

[27] Holmes ML Huntington ND Thong RP Brady J Hayakawa Y Andoniou CE. Peripheral Natural Killer Cell Maturation Depends on the Transcription Factor Aiolos. EMBO J (2014) 33(22):2721–34. doi: 10.15252/embj.201487900 PMC4282578 25319415

[28] Basar R Daher M Rezvani K. Next-Generation Cell Therapies: The Emerging Role of CAR-NK Cells. Blood Adv (2020) 4(22):5868–76. doi: 10.1182/bloodadvances.2020002547 PMC7686910 33232480

[29] Liu E Marin D Banerjee P Macapinlac HA Thompson P Basar R. Use of CAR-Transduced Natural Killer Cells in CD19-Positive Lymphoid Tumors. N Engl J Med (2020) 382(6):545–53. doi: 10.1056/NEJMoa1910607 PMC7101242 32023374

[30] Hernandez-Caselles T Miguel RC Ruiz-Alcaraz AJ Garcia-Penarrubia P. CD33 (Siglec-3) Inhibitory Function: Role in the NKG2D/DAP10 Activating Pathway. J Immunol Res (2019) 2019:6032141. doi: 10.1155/2019/6032141 31143782 PMC6501159

[31] Manser AR Weinhold S Uhrberg M. Human KIR Repertoires: Shaped by Genetic Diversity and Evolution. Immunol Rev (2015) 267(1):178–96. doi: 10.1111/imr.12316 26284478

[32] Barrow AD Trowsdale J. The Extended Human Leukocyte Receptor Complex: Diverse Ways of Modulating Immune Responses. Immunol Rev (2008) 224:98–123. doi: 10.1111/j.1600-065X.2008.00653.x 18759923

