# Supplementary material for: CD33 Delineates Two Functionally Distinct NK Cell Populations Divergent in Cytokine Production and Antibody-Mediated Cellular Cytotoxicity
Source: Front Immunol. 2022 Jan 4;12:798087. doi: 10.3389/fimmu.2021.798087 (PMC8764454; doi:10.3389/fimmu.2021.798087)
Supplement: Supplementary file 1 [file Presentation_1.pptx]

## Slide 1
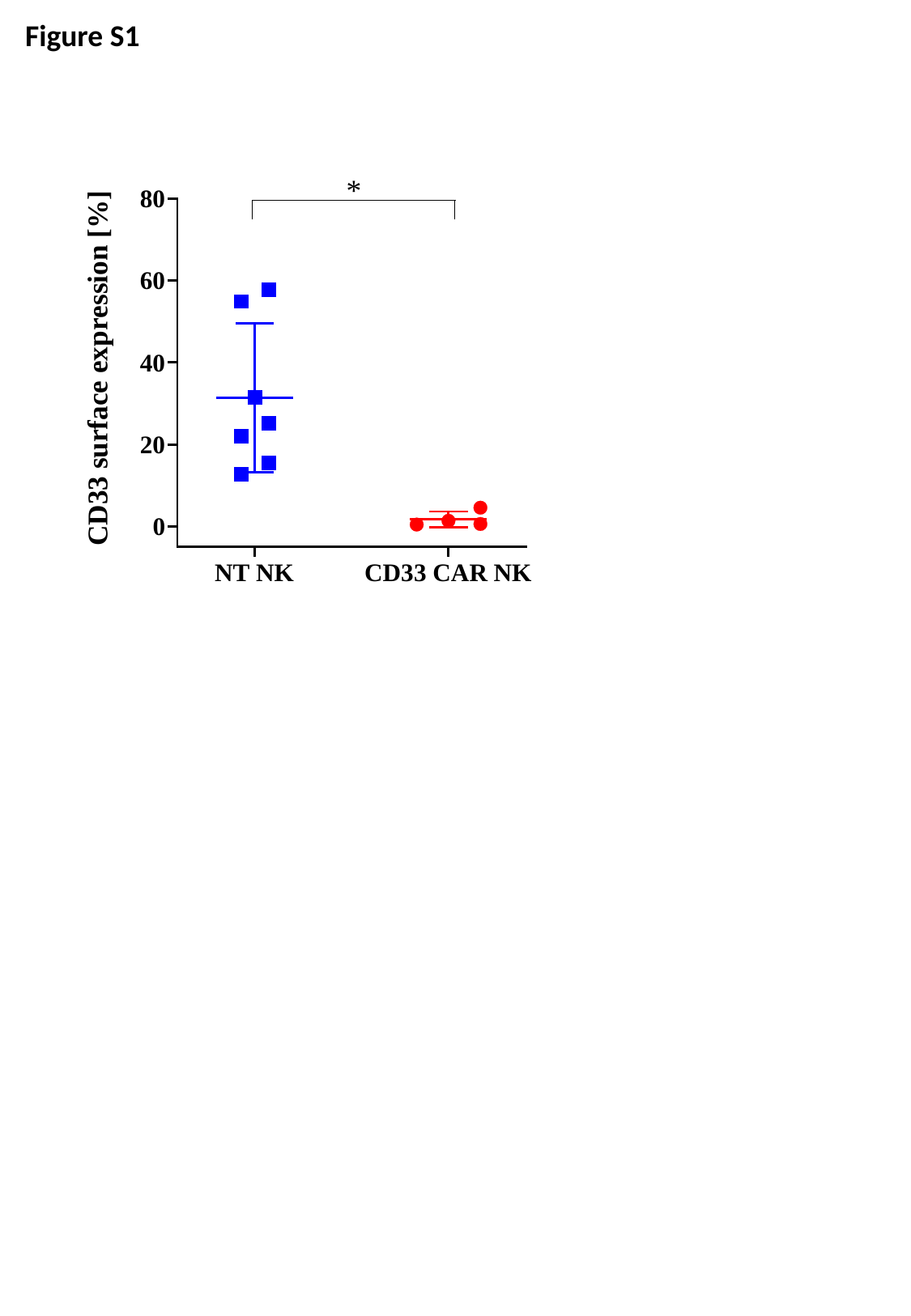

Figure S1
*

## Slide 2
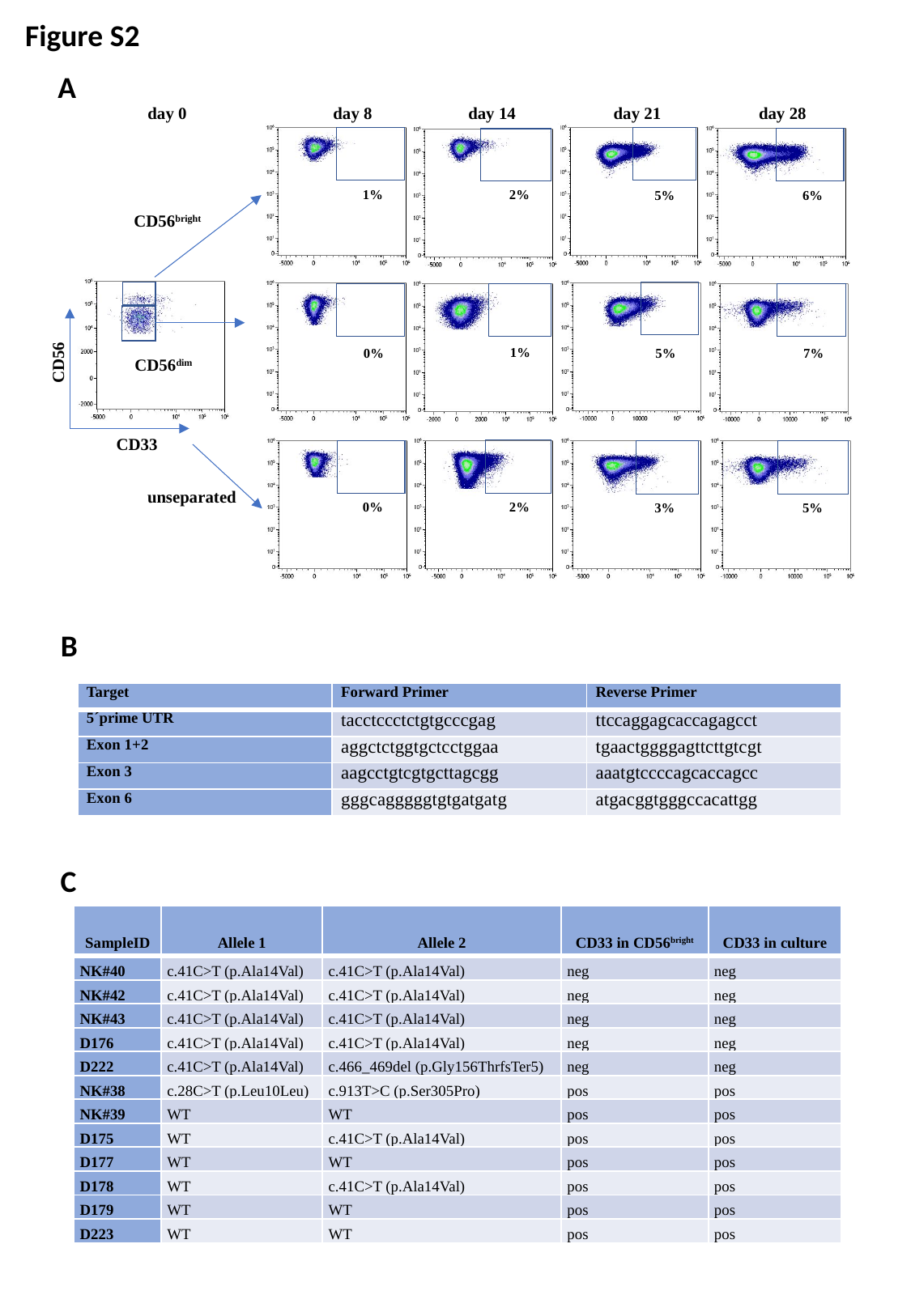

Figure S2
A
day 14
day 21
day 0
day 8
day 28
2%
1%
6%
5%
CD56bright
1%
0%
7%
5%
CD56
CD56dim
CD33
unseparated
2%
0%
5%
3%
B
| Target | Forward Primer | Reverse Primer |
| --- | --- | --- |
| 5´prime UTR | tacctccctctgtgcccgag | ttccaggagcaccagagcct |
| Exon 1+2 | aggctctggtgctcctggaa | tgaactggggagttcttgtcgt |
| Exon 3 | aagcctgtcgtgcttagcgg | aaatgtccccagcaccagcc |
| Exon 6 | gggcagggggtgtgatgatg | atgacggtgggccacattgg |
C
| SampleID | Allele 1 | Allele 2 | CD33 in CD56bright | CD33 in culture |
| --- | --- | --- | --- | --- |
| NK#40 | c.41C>T (p.Ala14Val) | c.41C>T (p.Ala14Val) | neg | neg |
| NK#42 | c.41C>T (p.Ala14Val) | c.41C>T (p.Ala14Val) | neg | neg |
| NK#43 | c.41C>T (p.Ala14Val) | c.41C>T (p.Ala14Val) | neg | neg |
| D176 | c.41C>T (p.Ala14Val) | c.41C>T (p.Ala14Val) | neg | neg |
| D222 | c.41C>T (p.Ala14Val) | c.466\_469del (p.Gly156ThrfsTer5) | neg | neg |
| NK#38 | c.28C>T (p.Leu10Leu) | c.913T>C (p.Ser305Pro) | pos | pos |
| NK#39 | WT | WT | pos | pos |
| D175 | WT | c.41C>T (p.Ala14Val) | pos | pos |
| D177 | WT | WT | pos | pos |
| D178 | WT | c.41C>T (p.Ala14Val) | pos | pos |
| D179 | WT | WT | pos | pos |
| D223 | WT | WT | pos | pos |

## Slide 3
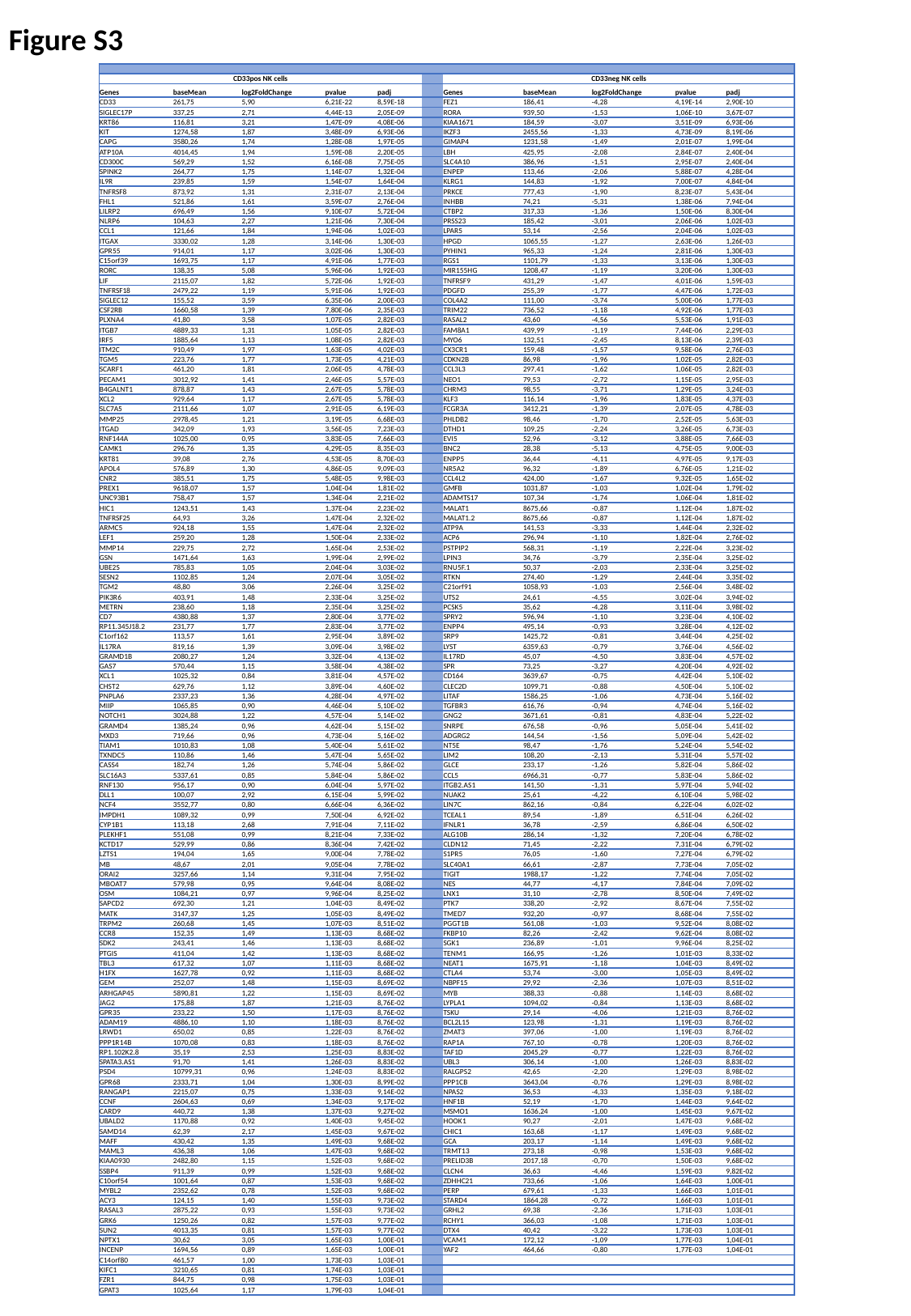

Figure S3
| | | | | | | | | | | |
| --- | --- | --- | --- | --- | --- | --- | --- | --- | --- | --- |
| CD33pos NK cells | | | | | | CD33neg NK cells | | | | |
| Genes | baseMean | log2FoldChange | pvalue | padj | | Genes | baseMean | log2FoldChange | pvalue | padj |
| CD33 | 261,75 | 5,90 | 6,21E-22 | 8,59E-18 | | FEZ1 | 186,41 | -4,28 | 4,19E-14 | 2,90E-10 |
| SIGLEC17P | 337,25 | 2,71 | 4,44E-13 | 2,05E-09 | | RORA | 939,50 | -1,53 | 1,06E-10 | 3,67E-07 |
| KRT86 | 116,81 | 3,21 | 1,47E-09 | 4,08E-06 | | KIAA1671 | 184,59 | -3,07 | 3,51E-09 | 6,93E-06 |
| KIT | 1274,58 | 1,87 | 3,48E-09 | 6,93E-06 | | IKZF3 | 2455,56 | -1,33 | 4,73E-09 | 8,19E-06 |
| CAPG | 3580,26 | 1,74 | 1,28E-08 | 1,97E-05 | | GIMAP4 | 1231,58 | -1,49 | 2,01E-07 | 1,99E-04 |
| ATP10A | 4014,45 | 1,94 | 1,59E-08 | 2,20E-05 | | LBH | 425,95 | -2,08 | 2,84E-07 | 2,40E-04 |
| CD300C | 569,29 | 1,52 | 6,16E-08 | 7,75E-05 | | SLC4A10 | 386,96 | -1,51 | 2,95E-07 | 2,40E-04 |
| SPINK2 | 264,77 | 1,75 | 1,14E-07 | 1,32E-04 | | ENPEP | 113,46 | -2,06 | 5,88E-07 | 4,28E-04 |
| IL9R | 239,85 | 1,59 | 1,54E-07 | 1,64E-04 | | KLRG1 | 144,83 | -1,92 | 7,00E-07 | 4,84E-04 |
| TNFRSF8 | 873,92 | 1,31 | 2,31E-07 | 2,13E-04 | | PRKCE | 777,43 | -1,90 | 8,23E-07 | 5,43E-04 |
| FHL1 | 521,86 | 1,61 | 3,59E-07 | 2,76E-04 | | INHBB | 74,21 | -5,31 | 1,38E-06 | 7,94E-04 |
| LILRP2 | 696,49 | 1,56 | 9,10E-07 | 5,72E-04 | | CTBP2 | 317,33 | -1,36 | 1,50E-06 | 8,30E-04 |
| NLRP6 | 104,63 | 2,27 | 1,21E-06 | 7,30E-04 | | PRSS23 | 185,42 | -3,01 | 2,06E-06 | 1,02E-03 |
| CCL1 | 121,66 | 1,84 | 1,94E-06 | 1,02E-03 | | LPAR5 | 53,14 | -2,56 | 2,04E-06 | 1,02E-03 |
| ITGAX | 3330,02 | 1,28 | 3,14E-06 | 1,30E-03 | | HPGD | 1065,55 | -1,27 | 2,63E-06 | 1,26E-03 |
| GPR55 | 914,01 | 1,17 | 3,02E-06 | 1,30E-03 | | PYHIN1 | 965,33 | -1,24 | 2,81E-06 | 1,30E-03 |
| C15orf39 | 1693,75 | 1,17 | 4,91E-06 | 1,77E-03 | | RGS1 | 1101,79 | -1,33 | 3,13E-06 | 1,30E-03 |
| RORC | 138,35 | 5,08 | 5,96E-06 | 1,92E-03 | | MIR155HG | 1208,47 | -1,19 | 3,20E-06 | 1,30E-03 |
| LIF | 2115,07 | 1,82 | 5,72E-06 | 1,92E-03 | | TNFRSF9 | 431,29 | -1,47 | 4,01E-06 | 1,59E-03 |
| TNFRSF18 | 2479,22 | 1,19 | 5,91E-06 | 1,92E-03 | | PDGFD | 255,39 | -1,77 | 4,47E-06 | 1,72E-03 |
| SIGLEC12 | 155,52 | 3,59 | 6,35E-06 | 2,00E-03 | | COL4A2 | 111,00 | -3,74 | 5,00E-06 | 1,77E-03 |
| CSF2RB | 1660,58 | 1,39 | 7,80E-06 | 2,35E-03 | | TRIM22 | 736,52 | -1,18 | 4,92E-06 | 1,77E-03 |
| PLXNA4 | 41,80 | 3,58 | 1,07E-05 | 2,82E-03 | | RASAL2 | 43,60 | -4,56 | 5,53E-06 | 1,91E-03 |
| ITGB7 | 4889,33 | 1,31 | 1,05E-05 | 2,82E-03 | | FAM8A1 | 439,99 | -1,19 | 7,44E-06 | 2,29E-03 |
| IRF5 | 1885,64 | 1,13 | 1,08E-05 | 2,82E-03 | | MYO6 | 132,51 | -2,45 | 8,13E-06 | 2,39E-03 |
| ITM2C | 910,49 | 1,97 | 1,63E-05 | 4,02E-03 | | CX3CR1 | 159,48 | -1,57 | 9,58E-06 | 2,76E-03 |
| TGM5 | 223,76 | 1,77 | 1,73E-05 | 4,21E-03 | | CDKN2B | 86,98 | -1,96 | 1,02E-05 | 2,82E-03 |
| SCARF1 | 461,20 | 1,81 | 2,06E-05 | 4,78E-03 | | CCL3L3 | 297,41 | -1,62 | 1,06E-05 | 2,82E-03 |
| PECAM1 | 3012,92 | 1,41 | 2,46E-05 | 5,57E-03 | | NEO1 | 79,53 | -2,72 | 1,15E-05 | 2,95E-03 |
| B4GALNT1 | 878,87 | 1,43 | 2,67E-05 | 5,78E-03 | | CHRM3 | 98,55 | -3,71 | 1,29E-05 | 3,24E-03 |
| XCL2 | 929,64 | 1,17 | 2,67E-05 | 5,78E-03 | | KLF3 | 116,14 | -1,96 | 1,83E-05 | 4,37E-03 |
| SLC7A5 | 2111,66 | 1,07 | 2,91E-05 | 6,19E-03 | | FCGR3A | 3412,21 | -1,39 | 2,07E-05 | 4,78E-03 |
| MMP25 | 2978,45 | 1,21 | 3,19E-05 | 6,68E-03 | | PHLDB2 | 98,46 | -1,70 | 2,52E-05 | 5,63E-03 |
| ITGAD | 342,09 | 1,93 | 3,56E-05 | 7,23E-03 | | DTHD1 | 109,25 | -2,24 | 3,26E-05 | 6,73E-03 |
| RNF144A | 1025,00 | 0,95 | 3,83E-05 | 7,66E-03 | | EVI5 | 52,96 | -3,12 | 3,88E-05 | 7,66E-03 |
| CAMK1 | 296,76 | 1,35 | 4,29E-05 | 8,35E-03 | | BNC2 | 28,38 | -5,13 | 4,75E-05 | 9,00E-03 |
| KRT81 | 39,08 | 2,76 | 4,53E-05 | 8,70E-03 | | ENPP5 | 36,44 | -4,11 | 4,97E-05 | 9,17E-03 |
| APOL4 | 576,89 | 1,30 | 4,86E-05 | 9,09E-03 | | NR5A2 | 96,32 | -1,89 | 6,76E-05 | 1,21E-02 |
| CNR2 | 385,51 | 1,75 | 5,48E-05 | 9,98E-03 | | CCL4L2 | 424,00 | -1,67 | 9,32E-05 | 1,65E-02 |
| PREX1 | 9618,07 | 1,57 | 1,04E-04 | 1,81E-02 | | GMFB | 1031,87 | -1,03 | 1,02E-04 | 1,79E-02 |
| UNC93B1 | 758,47 | 1,57 | 1,34E-04 | 2,21E-02 | | ADAMTS17 | 107,34 | -1,74 | 1,06E-04 | 1,81E-02 |
| HIC1 | 1243,51 | 1,43 | 1,37E-04 | 2,23E-02 | | MALAT1 | 8675,66 | -0,87 | 1,12E-04 | 1,87E-02 |
| TNFRSF25 | 64,93 | 3,26 | 1,47E-04 | 2,32E-02 | | MALAT1.2 | 8675,66 | -0,87 | 1,12E-04 | 1,87E-02 |
| ARMC5 | 924,18 | 1,55 | 1,47E-04 | 2,32E-02 | | ATP9A | 141,53 | -3,33 | 1,44E-04 | 2,32E-02 |
| LEF1 | 259,20 | 1,28 | 1,50E-04 | 2,33E-02 | | ACP6 | 296,94 | -1,10 | 1,82E-04 | 2,76E-02 |
| MMP14 | 229,75 | 2,72 | 1,65E-04 | 2,53E-02 | | PSTPIP2 | 568,31 | -1,19 | 2,22E-04 | 3,23E-02 |
| GSN | 1471,64 | 1,63 | 1,99E-04 | 2,99E-02 | | LPIN3 | 34,76 | -3,79 | 2,35E-04 | 3,25E-02 |
| UBE2S | 785,83 | 1,05 | 2,04E-04 | 3,03E-02 | | RNU5F.1 | 50,37 | -2,03 | 2,33E-04 | 3,25E-02 |
| SESN2 | 1102,85 | 1,24 | 2,07E-04 | 3,05E-02 | | RTKN | 274,40 | -1,29 | 2,44E-04 | 3,35E-02 |
| TGM2 | 48,80 | 3,06 | 2,26E-04 | 3,25E-02 | | C21orf91 | 1058,93 | -1,03 | 2,56E-04 | 3,48E-02 |
| PIK3R6 | 403,91 | 1,48 | 2,33E-04 | 3,25E-02 | | UTS2 | 24,61 | -4,55 | 3,02E-04 | 3,94E-02 |
| METRN | 238,60 | 1,18 | 2,35E-04 | 3,25E-02 | | PCSK5 | 35,62 | -4,28 | 3,11E-04 | 3,98E-02 |
| CD7 | 4380,88 | 1,37 | 2,80E-04 | 3,77E-02 | | SPRY2 | 596,94 | -1,10 | 3,23E-04 | 4,10E-02 |
| RP11.345J18.2 | 231,77 | 1,77 | 2,83E-04 | 3,77E-02 | | ENPP4 | 495,14 | -0,93 | 3,28E-04 | 4,12E-02 |
| C1orf162 | 113,57 | 1,61 | 2,95E-04 | 3,89E-02 | | SRP9 | 1425,72 | -0,81 | 3,44E-04 | 4,25E-02 |
| IL17RA | 819,16 | 1,39 | 3,09E-04 | 3,98E-02 | | LYST | 6359,63 | -0,79 | 3,76E-04 | 4,56E-02 |
| GRAMD1B | 2080,27 | 1,24 | 3,32E-04 | 4,13E-02 | | IL17RD | 45,07 | -4,50 | 3,83E-04 | 4,57E-02 |
| GAS7 | 570,44 | 1,15 | 3,58E-04 | 4,38E-02 | | SPR | 73,25 | -3,27 | 4,20E-04 | 4,92E-02 |
| XCL1 | 1025,32 | 0,84 | 3,81E-04 | 4,57E-02 | | CD164 | 3639,67 | -0,75 | 4,42E-04 | 5,10E-02 |
| CHST2 | 629,76 | 1,12 | 3,89E-04 | 4,60E-02 | | CLEC2D | 1099,71 | -0,88 | 4,50E-04 | 5,10E-02 |
| PNPLA6 | 2337,23 | 1,36 | 4,28E-04 | 4,97E-02 | | LITAF | 1586,25 | -1,06 | 4,73E-04 | 5,16E-02 |
| MIIP | 1065,85 | 0,90 | 4,46E-04 | 5,10E-02 | | TGFBR3 | 616,76 | -0,94 | 4,74E-04 | 5,16E-02 |
| NOTCH1 | 3024,88 | 1,22 | 4,57E-04 | 5,14E-02 | | GNG2 | 3671,61 | -0,81 | 4,83E-04 | 5,22E-02 |
| GRAMD4 | 1385,24 | 0,96 | 4,62E-04 | 5,15E-02 | | SNRPE | 676,58 | -0,96 | 5,05E-04 | 5,41E-02 |
| MXD3 | 719,66 | 0,96 | 4,73E-04 | 5,16E-02 | | ADGRG2 | 144,54 | -1,56 | 5,09E-04 | 5,42E-02 |
| TIAM1 | 1010,83 | 1,08 | 5,40E-04 | 5,61E-02 | | NT5E | 98,47 | -1,76 | 5,24E-04 | 5,54E-02 |
| TXNDC5 | 110,86 | 1,46 | 5,47E-04 | 5,65E-02 | | LIM2 | 108,20 | -2,13 | 5,31E-04 | 5,57E-02 |
| CASS4 | 182,74 | 1,26 | 5,74E-04 | 5,86E-02 | | GLCE | 233,17 | -1,26 | 5,82E-04 | 5,86E-02 |
| SLC16A3 | 5337,61 | 0,85 | 5,84E-04 | 5,86E-02 | | CCL5 | 6966,31 | -0,77 | 5,83E-04 | 5,86E-02 |
| RNF130 | 956,17 | 0,90 | 6,04E-04 | 5,97E-02 | | ITGB2.AS1 | 141,50 | -1,31 | 5,97E-04 | 5,94E-02 |
| DLL1 | 100,07 | 2,92 | 6,15E-04 | 5,99E-02 | | NUAK2 | 25,61 | -4,22 | 6,10E-04 | 5,98E-02 |
| NCF4 | 3552,77 | 0,80 | 6,66E-04 | 6,36E-02 | | LIN7C | 862,16 | -0,84 | 6,22E-04 | 6,02E-02 |
| IMPDH1 | 1089,32 | 0,99 | 7,50E-04 | 6,92E-02 | | TCEAL1 | 89,54 | -1,89 | 6,51E-04 | 6,26E-02 |
| CYP1B1 | 113,18 | 2,68 | 7,91E-04 | 7,11E-02 | | IFNLR1 | 36,78 | -2,59 | 6,86E-04 | 6,50E-02 |
| PLEKHF1 | 551,08 | 0,99 | 8,21E-04 | 7,33E-02 | | ALG10B | 286,14 | -1,32 | 7,20E-04 | 6,78E-02 |
| KCTD17 | 529,99 | 0,86 | 8,36E-04 | 7,42E-02 | | CLDN12 | 71,45 | -2,22 | 7,31E-04 | 6,79E-02 |
| LZTS1 | 194,04 | 1,65 | 9,00E-04 | 7,78E-02 | | S1PR5 | 76,05 | -1,60 | 7,27E-04 | 6,79E-02 |
| MB | 48,67 | 2,01 | 9,05E-04 | 7,78E-02 | | SLC40A1 | 66,61 | -2,87 | 7,73E-04 | 7,05E-02 |
| ORAI2 | 3257,66 | 1,14 | 9,31E-04 | 7,95E-02 | | TIGIT | 1988,17 | -1,22 | 7,74E-04 | 7,05E-02 |
| MBOAT7 | 579,98 | 0,95 | 9,64E-04 | 8,08E-02 | | NES | 44,77 | -4,17 | 7,84E-04 | 7,09E-02 |
| OSM | 1084,21 | 0,97 | 9,96E-04 | 8,25E-02 | | LNX1 | 31,10 | -2,78 | 8,50E-04 | 7,49E-02 |
| SAPCD2 | 692,30 | 1,21 | 1,04E-03 | 8,49E-02 | | PTK7 | 338,20 | -2,92 | 8,67E-04 | 7,55E-02 |
| MATK | 3147,37 | 1,25 | 1,05E-03 | 8,49E-02 | | TMED7 | 932,20 | -0,97 | 8,68E-04 | 7,55E-02 |
| TRPM2 | 260,68 | 1,45 | 1,07E-03 | 8,51E-02 | | PGGT1B | 561,08 | -1,03 | 9,52E-04 | 8,08E-02 |
| CCR8 | 152,35 | 1,49 | 1,13E-03 | 8,68E-02 | | FKBP10 | 82,26 | -2,42 | 9,62E-04 | 8,08E-02 |
| SDK2 | 243,41 | 1,46 | 1,13E-03 | 8,68E-02 | | SGK1 | 236,89 | -1,01 | 9,96E-04 | 8,25E-02 |
| PTGIS | 411,04 | 1,42 | 1,13E-03 | 8,68E-02 | | TENM1 | 166,95 | -1,26 | 1,01E-03 | 8,33E-02 |
| TBL3 | 617,32 | 1,07 | 1,11E-03 | 8,68E-02 | | NEAT1 | 1675,91 | -1,18 | 1,04E-03 | 8,49E-02 |
| H1FX | 1627,78 | 0,92 | 1,11E-03 | 8,68E-02 | | CTLA4 | 53,74 | -3,00 | 1,05E-03 | 8,49E-02 |
| GEM | 252,07 | 1,48 | 1,15E-03 | 8,69E-02 | | NBPF15 | 29,92 | -2,36 | 1,07E-03 | 8,51E-02 |
| ARHGAP45 | 5890,81 | 1,22 | 1,15E-03 | 8,69E-02 | | MYB | 388,33 | -0,88 | 1,14E-03 | 8,68E-02 |
| JAG2 | 175,88 | 1,87 | 1,21E-03 | 8,76E-02 | | LYPLA1 | 1094,02 | -0,84 | 1,13E-03 | 8,68E-02 |
| GPR35 | 233,22 | 1,50 | 1,17E-03 | 8,76E-02 | | TSKU | 29,14 | -4,06 | 1,21E-03 | 8,76E-02 |
| ADAM19 | 4886,10 | 1,10 | 1,18E-03 | 8,76E-02 | | BCL2L15 | 123,98 | -1,31 | 1,19E-03 | 8,76E-02 |
| LRWD1 | 650,02 | 0,85 | 1,22E-03 | 8,76E-02 | | ZMAT3 | 397,06 | -1,00 | 1,19E-03 | 8,76E-02 |
| PPP1R14B | 1070,08 | 0,83 | 1,18E-03 | 8,76E-02 | | RAP1A | 767,10 | -0,78 | 1,20E-03 | 8,76E-02 |
| RP1.102K2.8 | 35,19 | 2,53 | 1,25E-03 | 8,83E-02 | | TAF1D | 2045,29 | -0,77 | 1,22E-03 | 8,76E-02 |
| SPATA3.AS1 | 91,70 | 1,41 | 1,26E-03 | 8,83E-02 | | UBL3 | 306,14 | -1,00 | 1,26E-03 | 8,83E-02 |
| PSD4 | 10799,31 | 0,96 | 1,24E-03 | 8,83E-02 | | RALGPS2 | 42,65 | -2,20 | 1,29E-03 | 8,98E-02 |
| GPR68 | 2333,71 | 1,04 | 1,30E-03 | 8,99E-02 | | PPP1CB | 3643,04 | -0,76 | 1,29E-03 | 8,98E-02 |
| RANGAP1 | 2215,07 | 0,75 | 1,33E-03 | 9,14E-02 | | NPAS2 | 36,53 | -4,33 | 1,35E-03 | 9,18E-02 |
| CCNF | 2604,63 | 0,69 | 1,34E-03 | 9,17E-02 | | HNF1B | 52,19 | -1,70 | 1,44E-03 | 9,64E-02 |
| CARD9 | 440,72 | 1,38 | 1,37E-03 | 9,27E-02 | | MSMO1 | 1636,24 | -1,00 | 1,45E-03 | 9,67E-02 |
| UBALD2 | 1170,88 | 0,92 | 1,40E-03 | 9,45E-02 | | HOOK1 | 90,27 | -2,01 | 1,47E-03 | 9,68E-02 |
| SAMD14 | 62,39 | 2,17 | 1,45E-03 | 9,67E-02 | | CHIC1 | 163,68 | -1,17 | 1,49E-03 | 9,68E-02 |
| MAFF | 430,42 | 1,35 | 1,49E-03 | 9,68E-02 | | GCA | 203,17 | -1,14 | 1,49E-03 | 9,68E-02 |
| MAML3 | 436,38 | 1,06 | 1,47E-03 | 9,68E-02 | | TRMT13 | 273,18 | -0,98 | 1,53E-03 | 9,68E-02 |
| KIAA0930 | 2482,80 | 1,15 | 1,52E-03 | 9,68E-02 | | PRELID3B | 2017,18 | -0,70 | 1,50E-03 | 9,68E-02 |
| SSBP4 | 911,39 | 0,99 | 1,52E-03 | 9,68E-02 | | CLCN4 | 36,63 | -4,46 | 1,59E-03 | 9,82E-02 |
| C10orf54 | 1001,64 | 0,87 | 1,53E-03 | 9,68E-02 | | ZDHHC21 | 733,66 | -1,06 | 1,64E-03 | 1,00E-01 |
| MYBL2 | 2352,62 | 0,78 | 1,52E-03 | 9,68E-02 | | PERP | 679,61 | -1,33 | 1,66E-03 | 1,01E-01 |
| ACY3 | 124,15 | 1,40 | 1,55E-03 | 9,73E-02 | | STARD4 | 1864,28 | -0,72 | 1,66E-03 | 1,01E-01 |
| RASAL3 | 2875,22 | 0,93 | 1,55E-03 | 9,73E-02 | | GRHL2 | 69,38 | -2,36 | 1,71E-03 | 1,03E-01 |
| GRK6 | 1250,26 | 0,82 | 1,57E-03 | 9,77E-02 | | RCHY1 | 366,03 | -1,08 | 1,71E-03 | 1,03E-01 |
| SUN2 | 4013,35 | 0,81 | 1,57E-03 | 9,77E-02 | | DTX4 | 40,42 | -3,22 | 1,73E-03 | 1,03E-01 |
| NPTX1 | 30,62 | 3,05 | 1,65E-03 | 1,00E-01 | | VCAM1 | 172,12 | -1,09 | 1,77E-03 | 1,04E-01 |
| INCENP | 1694,56 | 0,89 | 1,65E-03 | 1,00E-01 | | YAF2 | 464,66 | -0,80 | 1,77E-03 | 1,04E-01 |
| C14orf80 | 461,57 | 1,00 | 1,73E-03 | 1,03E-01 | | | | | | |
| KIFC1 | 3210,65 | 0,81 | 1,74E-03 | 1,03E-01 | | | | | | |
| FZR1 | 844,75 | 0,98 | 1,75E-03 | 1,03E-01 | | | | | | |
| GPAT3 | 1025,64 | 1,17 | 1,79E-03 | 1,04E-01 | | | | | | |

## Slide 4
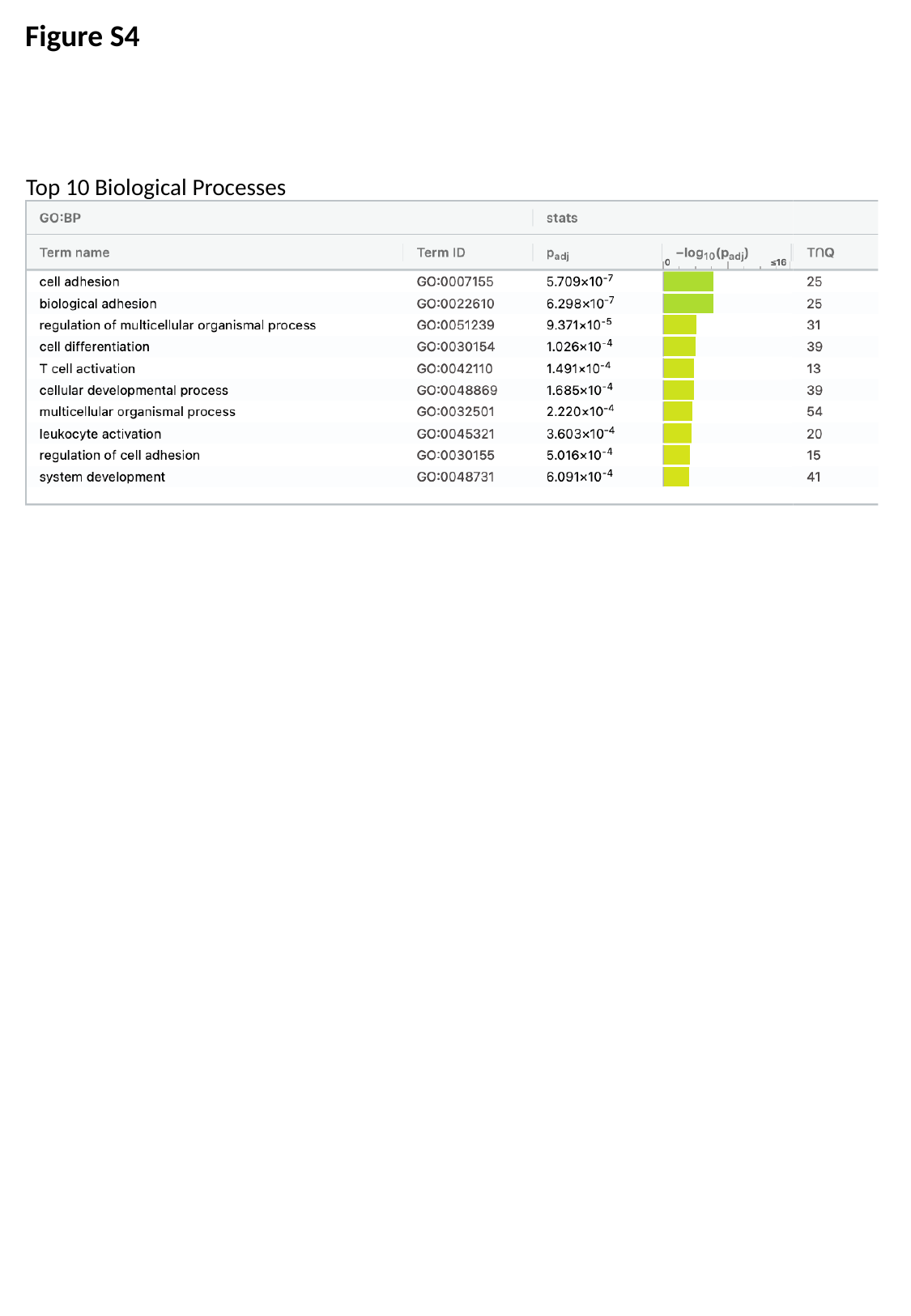

Figure S4
Top 10 Biological Processes

## Slide 5
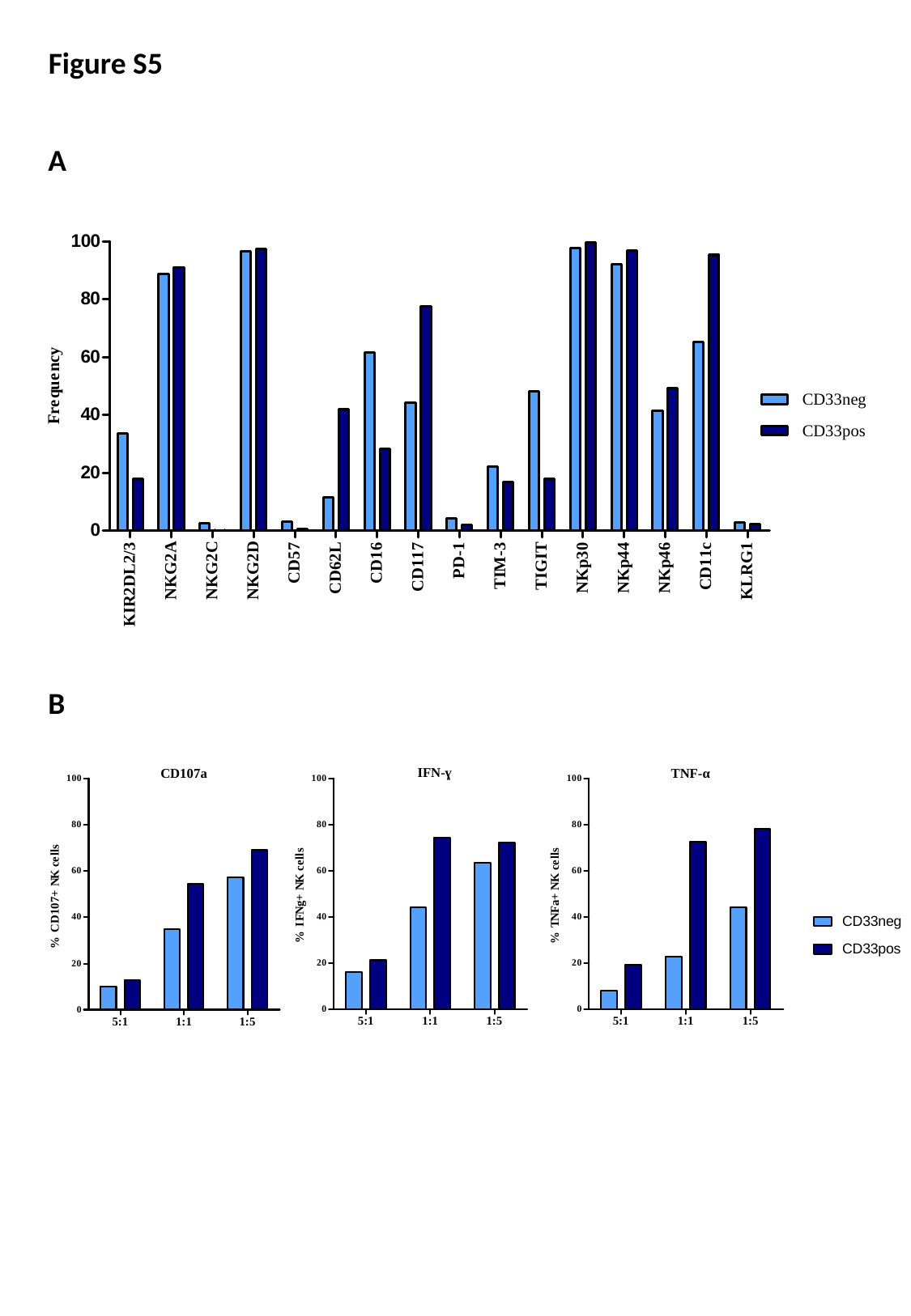

Figure S5
A
B
IFN-ɣ
CD107a
TNF-α

## Slide 6
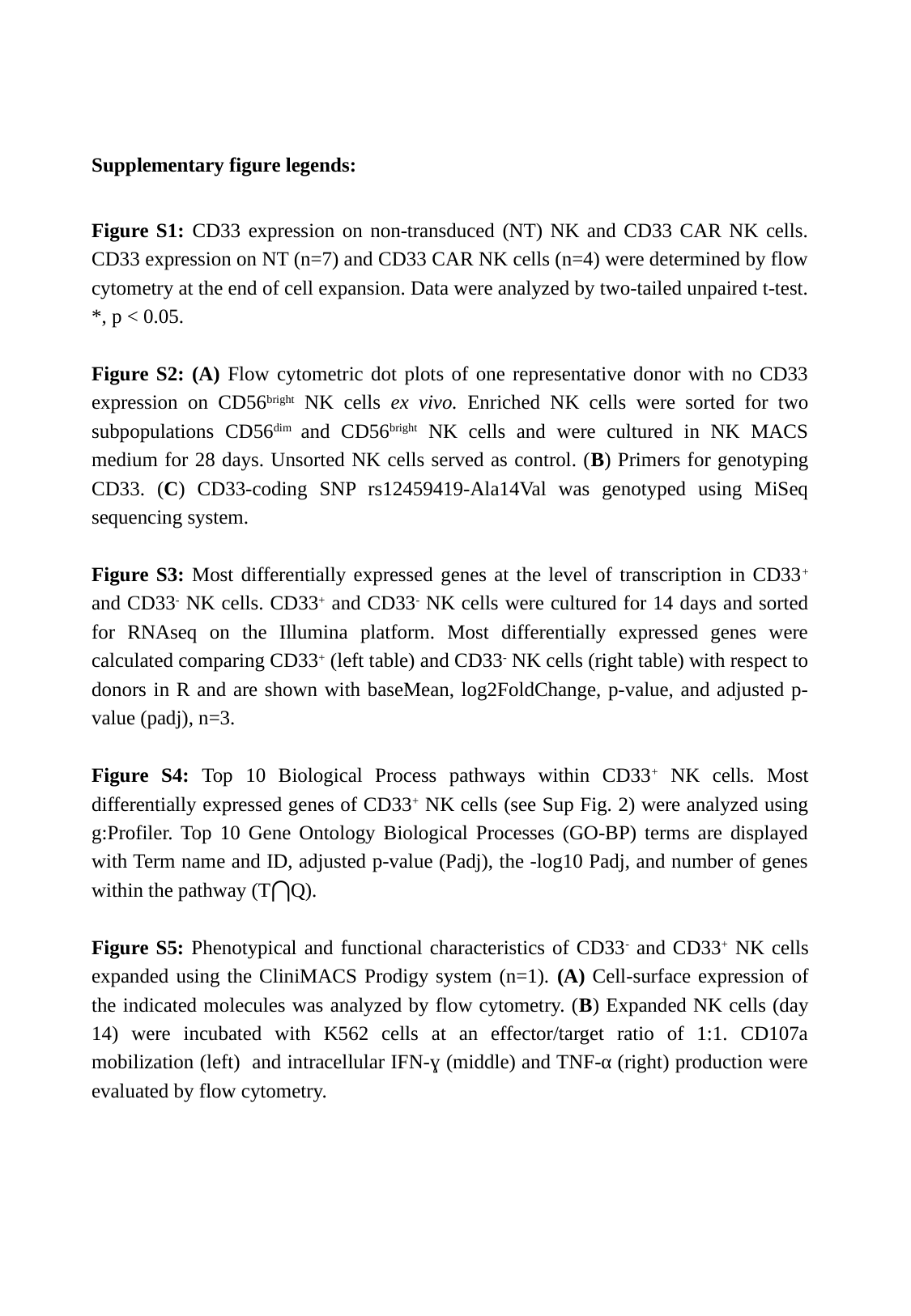

Supplementary figure legends:
Figure S1: CD33 expression on non-transduced (NT) NK and CD33 CAR NK cells. CD33 expression on NT (n=7) and CD33 CAR NK cells (n=4) were determined by flow cytometry at the end of cell expansion. Data were analyzed by two-tailed unpaired t-test. *, p < 0.05.
Figure S2: (A) Flow cytometric dot plots of one representative donor with no CD33 expression on CD56bright NK cells ex vivo. Enriched NK cells were sorted for two subpopulations CD56dim and CD56bright NK cells and were cultured in NK MACS medium for 28 days. Unsorted NK cells served as control. (B) Primers for genotyping CD33. (C) CD33-coding SNP rs12459419-Ala14Val was genotyped using MiSeq sequencing system.
Figure S3: Most differentially expressed genes at the level of transcription in CD33+ and CD33- NK cells. CD33+ and CD33- NK cells were cultured for 14 days and sorted for RNAseq on the Illumina platform. Most differentially expressed genes were calculated comparing CD33+ (left table) and CD33- NK cells (right table) with respect to donors in R and are shown with baseMean, log2FoldChange, p-value, and adjusted p-value (padj), n=3.
Figure S4: Top 10 Biological Process pathways within CD33+ NK cells. Most differentially expressed genes of CD33+ NK cells (see Sup Fig. 2) were analyzed using g:Profiler. Top 10 Gene Ontology Biological Processes (GO-BP) terms are displayed with Term name and ID, adjusted p-value (Padj), the -log10 Padj, and number of genes within the pathway (T⋂Q).
Figure S5: Phenotypical and functional characteristics of CD33- and CD33+ NK cells expanded using the CliniMACS Prodigy system (n=1). (A) Cell-surface expression of the indicated molecules was analyzed by flow cytometry. (B) Expanded NK cells (day 14) were incubated with K562 cells at an effector/target ratio of 1:1. CD107a mobilization (left) and intracellular IFN-ɣ (middle) and TNF-α (right) production were evaluated by flow cytometry.

## Slide 7
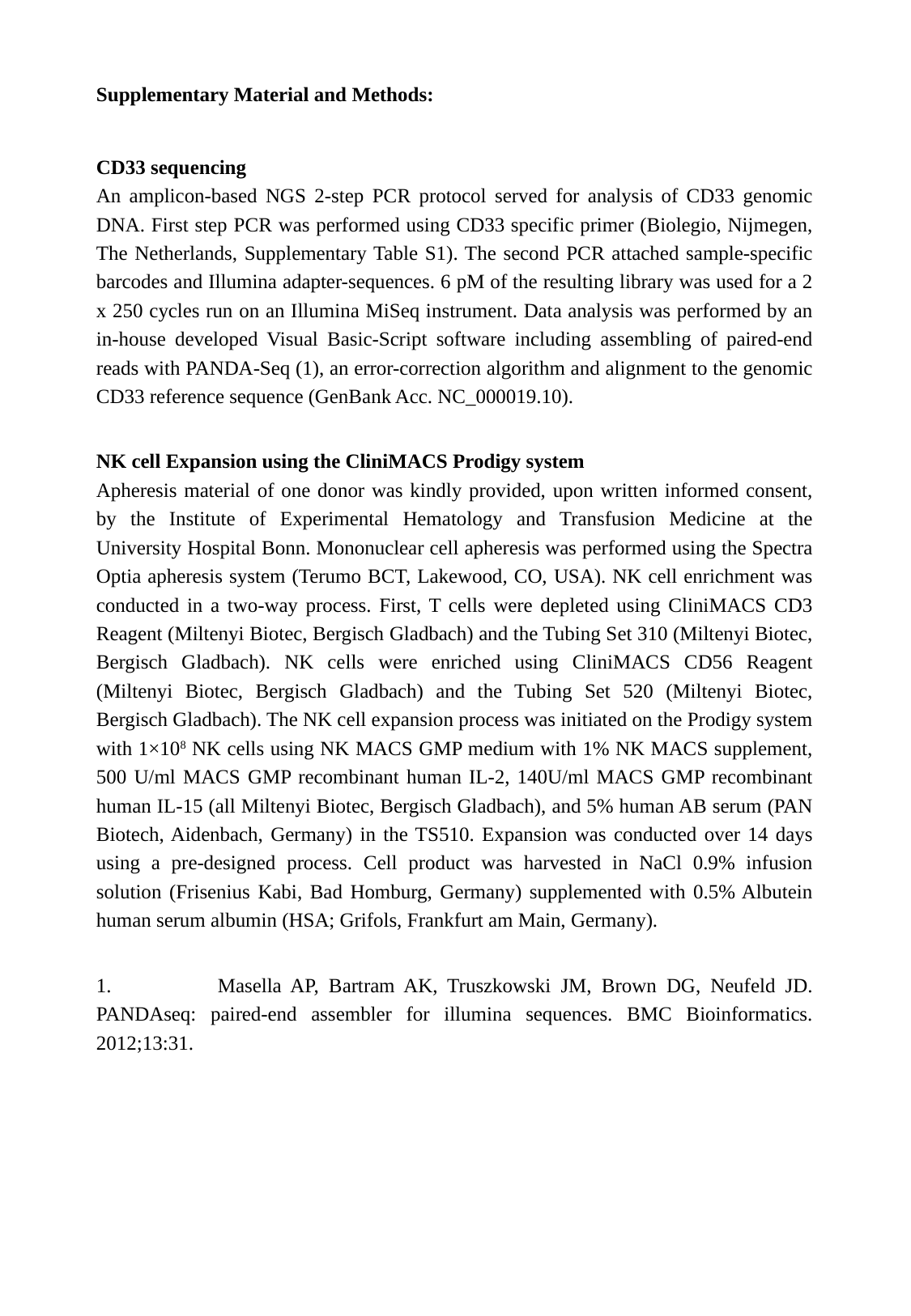

Supplementary Material and Methods:
CD33 sequencing
An amplicon-based NGS 2-step PCR protocol served for analysis of CD33 genomic DNA. First step PCR was performed using CD33 specific primer (Biolegio, Nijmegen, The Netherlands, Supplementary Table S1). The second PCR attached sample-specific barcodes and Illumina adapter-sequences. 6 pM of the resulting library was used for a 2 x 250 cycles run on an Illumina MiSeq instrument. Data analysis was performed by an in-house developed Visual Basic-Script software including assembling of paired-end reads with PANDA-Seq (1), an error-correction algorithm and alignment to the genomic CD33 reference sequence (GenBank Acc. NC_000019.10).
NK cell Expansion using the CliniMACS Prodigy system
Apheresis material of one donor was kindly provided, upon written informed consent, by the Institute of Experimental Hematology and Transfusion Medicine at the University Hospital Bonn. Mononuclear cell apheresis was performed using the Spectra Optia apheresis system (Terumo BCT, Lakewood, CO, USA). NK cell enrichment was conducted in a two-way process. First, T cells were depleted using CliniMACS CD3 Reagent (Miltenyi Biotec, Bergisch Gladbach) and the Tubing Set 310 (Miltenyi Biotec, Bergisch Gladbach). NK cells were enriched using CliniMACS CD56 Reagent (Miltenyi Biotec, Bergisch Gladbach) and the Tubing Set 520 (Miltenyi Biotec, Bergisch Gladbach). The NK cell expansion process was initiated on the Prodigy system with 1×10^8^ NK cells using NK MACS GMP medium with 1% NK MACS supplement, 500 U/ml MACS GMP recombinant human IL-2, 140U/ml MACS GMP recombinant human IL-15 (all Miltenyi Biotec, Bergisch Gladbach), and 5% human AB serum (PAN Biotech, Aidenbach, Germany) in the TS510. Expansion was conducted over 14 days using a pre-designed process. Cell product was harvested in NaCl 0.9% infusion solution (Frisenius Kabi, Bad Homburg, Germany) supplemented with 0.5% Albutein human serum albumin (HSA; Grifols, Frankfurt am Main, Germany).
1.	Masella AP, Bartram AK, Truszkowski JM, Brown DG, Neufeld JD. PANDAseq: paired-end assembler for illumina sequences. BMC Bioinformatics. 2012;13:31.
